# Advanced Glycation End Products: From Pathogenesis to Treatment

**DOI:** 10.1002/mco2.71073

**Published:** 2026-10-13

**Authors:** Tak‐Ho Lo, Junying Zou, Jingyi Zhang, Kaijun Lin, Minhui Zhu, Shuangqi Lu, Xin Liu, Mengyao Wu

**Affiliations:** ^1^ School of Life Sciences Guangzhou University Guangdong China; ^2^ Department of Health Technology and Informatics The Hong Kong Polytechnic University Hong Kong China; ^3^ College of Biology Hunan University Hunan China; ^4^ School of Chemistry and Chemical Engineering Guangzhou University Guangdong China

**Keywords:** advanced glycation end products, carbonyl stress, extracellular matrix crosslinking, glyoxalase, RAGE

## Abstract

Advanced glycation end products (AGEs) are chemically heterogeneous modifications generated through nonenzymatic glycation, glycoxidation, and carbonyl stress. Their accumulation increases during aging and is accelerated by hyperglycemia, oxidative stress, inflammation, exogenous exposure, and impaired renal clearance. Although AGE biology has often been framed around the AGE–RAGE–oxidative stress axis, current evidence supports a broader systems‐level model in which AGE burden reflects the balance among formation, detoxification, tissue retention, and clearance. This review summarizes AGE chemistry, endogenous and exogenous sources, detoxification pathways, receptor systems, and mechanisms of AGE‐mediated tissue injury. We distinguish two complementary pathogenic routes: receptor‐independent structural damage, including protein dysfunction, proteostatic stress, extracellular matrix crosslinking, and altered tissue mechanics; and receptor‐dependent signaling, mainly through RAGE‐mediated redox‐inflammatory amplification. We then examine the clinical relevance of AGEs in diabetes, cardiovascular disease, chronic kidney disease, neurovascular and retinal dysfunction, fibrosis, and aging‐related tissue decline. Particular attention is given to compartment‐specific biomarker interpretation, because circulating free adducts, protein‐bound AGEs, tissue autofluorescence, dietary AGE estimates, and dicarbonyl stress markers are not interchangeable. Finally, we evaluate therapeutic strategies and propose a framework that matches each intervention to the relevant mechanism and can be tested clinically. In doing so, the review clarifies major conceptual and methodological uncertainties in AGE research and provides a foundation for more rigorous biomarker interpretation, study design, and clinical translation.

## Graphical abstract

Therapeutic landscape of AGE‐targeted intervention. Schematic representation of intervention strategies targeting distinct stages of AGE biology. Upstream prevention aims to reduce exposure and formation through low‐AGE cooking, smoking cessation, glycemic control, and metabolic optimization. Formation inhibition and carbonyl trapping target reactive dicarbonyl intermediates such as MGO, GO, and 3‐DG before stable AGEs are formed. Detoxification and clearance enhancement aim to strengthen glyoxalase/GSH‐dependent defense, NRF2‐linked stress responses, renal clearance, and proteostatic turnover. Matrix‐targeted approaches address tissue accumulation and crosslinking by limiting new crosslink formation, promoting matrix remodeling, or restoring tissue compliance. AGE–RAGE‐targeted strategies include RAGE antagonism, sRAGE or decoy approaches, RAGE–DIAPH1 blockade, and downstream pathway inhibition. Natural compounds may act across several nodes by reducing dicarbonyl stress, suppressing AGE–RAGE inflammation, and protecting matrix or barrier integrity. Effective translation requires matching each intervention to the relevant AGE species, compartment, disease stage, and functional endpoint. *Abbreviations*: 3‐DG, 3‐deoxyglucosone; AGE, advanced glycation end product; DIAPH1, diaphanous‐related formin 1; GO, glyoxal; GSH, reduced glutathione; MGO, methylglyoxal; NRF2, nuclear factor erythroid 2‐related factor 2; RAGE, receptor for advanced glycation end products; sRAGE, soluble receptor for advanced glycation end products.

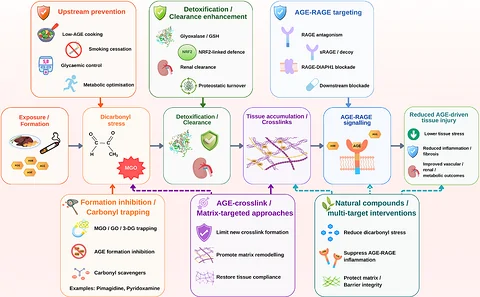

## 1 Introduction

Advanced glycation end products (AGEs) comprise a chemically heterogeneous group of largely irreversible adducts generated through nonenzymatic glycation, glycoxidation, and carbonyl stress reactions involving proteins, lipids, and nucleic acids. The biochemical origin of this field can be traced to Maillard's early description of amino acid–sugar reactions in 1912 and Hodge's later formalization of nonenzymatic browning chemistry in food systems [1]. Since then, AGE biology has expanded from food chemistry and diabetic biochemistry into a broader framework for understanding chronic metabolic, vascular, renal, inflammatory, and degenerative diseases [2, 3]. AGE accumulation occurs gradually during aging but is accelerated under conditions of persistent hyperglycemia, oxidative stress, lipid peroxidation, chronic inflammation, and impaired renal function [4, 5]. In recent years, the field has shifted from viewing AGEs as a general class of glycation products toward more chemically and biologically defined entities, particularly reactive dicarbonyl‐derived adducts generated from methylglyoxal, glyoxal, and 3‐deoxyglucosone [6, 7, 8, 9]. Methylglyoxal is especially relevant because it links increased glycolytic flux, glyoxalase‐dependent detoxification, arginine‐ and lysine‐derived adduct formation, and vascular or renal complication risk [10]. Exogenous AGE exposure from thermally processed foods and tobacco smoke also remains relevant, but systemic AGE burden cannot be inferred from exposure alone because digestion, absorption, microbiota metabolism, epithelial responses, tissue turnover, and renal clearance all influence biological availability [11, 12, 13]. Thus, AGE burden is best conceptualized as a dynamic balance among endogenous formation, environmental exposure, detoxification, tissue retention, and clearance.

The clinical relevance of AGEs is most firmly established in diabetes and its complications, where hyperglycemia‐driven dicarbonyl stress contributes to microvascular and macrovascular injury [14, 15]. However, recent advances have broadened the field beyond the conventional paradigm centered on AGE signaling through the receptor for AGEs (RAGE) and oxidative stress. First, methylglyoxal and its DNA, RNA, and protein adducts are increasingly recognized as disease‐associated molecular species rather than nonspecific by‐products of hyperglycemia; notably, urinary methylglyoxal adducts have been reported as prognostic biomarkers for diabetic kidney disease in patients with Type 1 diabetes [16, 17]. Second, advances in mass spectrometry and tissue‐level matrix analysis have renewed interest in receptor‐independent AGE pathology, especially extracellular matrix crosslinking, tissue stiffening, and impaired matrix remodeling. In scarred liver tissue, AGE‐mediated matrix crosslinking altered collagen biomechanics and macrophage remodeling responses, supporting the view that AGEs can drive disease through structural and mechanical mechanisms independent of canonical receptor signaling [18]. Third, RAGE biology has become more therapeutically nuanced. Beyond the conventional view of RAGE as an extracellular AGE receptor, recent work has highlighted intracellular RAGE signaling through diaphanous‐related formin 1 (DIAPH1) as a druggable checkpoint that links AGE/RAGE activation to cytoskeletal remodeling, inflammation, vascular injury, and wound healing impairment [19, 20]. Finally, chemical‐biology studies have emphasized that protein glycation operates through a diverse molecular “language,” meaning that total AGE fluorescence or bulk immunoreactivity may not capture the pathogenic species most relevant to tissue injury or therapeutic response [21]. These developments indicate that AGE research is entering a more molecularly specific and translationally demanding phase.

Despite this progress, the AGE literature remains fragmented across chemistry, biomarkers, disease‐specific mechanisms, and intervention studies. Many reviews continue to treat AGEs as a single pathological entity and repeatedly invoke AGE–RAGE signaling, oxidative stress, and inflammation without adequately distinguishing specific AGE species, biological compartments, or levels of evidence [22, 23]. This limits mechanistic clarity and weakens clinical translation, particularly because circulating AGEs, protein‐bound AGEs, free AGE adducts, tissue autofluorescence, dietary AGE estimates, and dicarbonyl stress markers are not interchangeable measurements [9, 12, 24]. In addition, receptor‐independent mechanisms such as protein dysfunction, impaired proteostasis, extracellular matrix crosslinking, and altered tissue mechanics are often insufficiently underintegrated despite their direct relevance to chronic tissue remodeling [5, 18]. To address these gaps, this article was developed as a narrative review. Relevant literature was identified through targeted searches of PubMed/MEDLINE and Web of Science up to June 2026, supplemented by backward and forward citation tracking of key reviews and primary studies. Search concepts combined “advanced glycation end products” and named AGE or dicarbonyl species, including methylglyoxal, glyoxal, Nε‐carboxymethyllysine (CML), Nε‐carboxyethyllysine (CEL), methylglyoxal‐derived hydroimidazolone (MG‐H1), and glucosepane, with terms relating to formation, detoxification, renal clearance, RAGE–DIAPH1 signaling, extracellular matrix crosslinking, biomarkers, disease outcomes, clinical trials, and therapeutic interventions. Study selection was guided by relevance to the review framework, chemical and methodological specificity, level of evidence, and recency. Priority was given to original mechanistic studies, longitudinal human studies, randomized or controlled clinical studies, and recent authoritative reviews, while older landmark studies were retained when they established foundational chemical or biological concepts. Therapeutic evidence was considered across cell‐free and cellular experiments, animal models, observational human studies, and interventional trials, with ClinicalTrials.gov consulted where registration information was available.

On this basis, the review examines AGE biology through an integrated framework linking formation, exposure, detoxification, clearance, tissue accumulation, molecular pathogenesis, disease relevance, biomarker interpretation, and therapeutic targetability. Diabetes complications are used as the central clinical anchor because they provide the strongest mechanistic and translational evidence base, while cardiovascular, renal, neurovascular, retinal, fibrotic, and aging‐related contexts are discussed comparatively. The review first summarizes AGE formation, exogenous exposure, detoxification, clearance, and receptor biology; then distinguishes receptor‐independent damage from receptor‐dependent signaling and convergent injury programs; next evaluates disease relevance and clinical assessment; and finally discusses prevention, pharmacologic intervention, natural compounds, and translational barriers. By emphasizing molecular specificity, compartment‐aware measurement, evidence maturity, and target engagement, this review aims to update the AGE field beyond broad associative descriptions and toward clinically testable models of AGE‐mediated chronic disease.

## 2 AGE Biology

AGE biology is best understood as a lifecycle rather than as a linear chemical process. This section first summarizes endogenous AGE formation through Maillard chemistry, glycoxidation, and dicarbonyl stress; then considers exogenous exposure from diet, smoking, and related environmental sources; next examines detoxification and clearance systems that determine whether AGE precursors and adducts persist; and finally discusses receptor‐mediated recognition as the entry point for cellular signaling. This sequence establishes the biochemical basis for the later distinction between AGE formation, tissue accumulation, receptor‐independent structural damage, and receptor‐dependent inflammatory amplification.

### 2.1 Endogenous Formation of AGEs

Endogenous AGE formation is initiated by nonenzymatic reactions between carbonyl groups and nucleophilic sites in biological macromolecules. In proteins, lysine and arginine residues are the principal glycation targets because their ε‐amino and guanidino groups are chemically reactive and frequently solvent‐accessible, although cysteine, histidine, phospholipids, and nucleic acid bases may also undergo carbonyl‐derived modification under conditions of increased carbonyl stress [25]. In the classical Maillard pathway, reducing sugars react with protein amino groups to form reversible Schiff bases, which undergo Amadori rearrangement into more stable early glycation products, such as fructosyl‐lysine [26]. Subsequent oxidation, dehydration, fragmentation, and rearrangement reactions then generate a chemically diverse spectrum of stable AGEs [27]. However, in vivo AGE formation is not determined by glucose concentration alone. It is shaped by oxidative status, transition metal‐catalyzed oxidation, lipid peroxidation, local protein turnover, tissue oxygenation, and detoxification capacity [28, 29]. Long‐lived proteins, including collagen, elastin, crystallins, and basement membrane proteins, are therefore especially vulnerable to cumulative glycation, because they persist long enough to undergo sequential early and advanced glycation reactions.

Reactive dicarbonyl intermediates provide a faster and biologically more reactive route to AGE formation. Methylglyoxal, glyoxal, and 3‐deoxyglucosone are the most widely studied intermediates in this process. Methylglyoxal is generated mainly through spontaneous degradation of triose phosphates during glycolysis, whereas glyoxal arises from glucose autoxidation, lipid peroxidation, and oxidative degradation of glycated proteins [30]. 3‐Deoxyglucosone is produced largely through degradation of Amadori products and sugar autoxidation [8]. Because these dicarbonyls react with lysine and arginine residues much more rapidly than glucose, dicarbonyl stress has become a more chemically precise explanation for accelerated AGE formation in diabetes, cardiometabolic disease, and aging‐related tissue dysfunction [31, 32]. This distinction matters analytically, because different AGE species reflect different chemical routes and biological compartments. CML can arise from glycoxidation and lipid peroxidation derived carbonyl chemistry, CEL is more closely linked to methylglyoxal‐mediated lysine modification, MG‐H1 is a major arginine‐derived adduct, pentosidine represents a fluorescent glycoxidative crosslink, and glucosepane is a major but analytically challenging collagen crosslink in long lived extracellular matrix [33, 34, 35]. Recent advances in targeted liquid chromatography–tandem mass spectrometry (LC–MS/MS) have therefore shifted the field from bulk or fluorescence‐based AGE estimation toward adduct‐specific profiling of free and protein‐bound AGE species [36]. This development is crucial because free AGE adducts may reflect recent formation, proteolytic release, diet‐derived absorption, or renal clearance, whereas protein‐bound and matrix‐bound AGEs integrate modification over longer time scales and may be more relevant to tissue stiffness and impaired remodeling. Endogenous AGE formation is therefore best viewed as a network of glycation, glycoxidation, dicarbonyl generation, macromolecular modification, and turnover.

### 2.2 Exogenous AGE Exposure

Exogenous AGEs are generated primarily during thermal processing of foods, in which reducing sugars and reactive carbonyls react with amino groups in proteins, peptides, and lipids through Maillard‐type chemistry. The extent of AGE formation depends on food composition, temperature, heating duration, pH, water activity, and processing method [37, 38]. Protein‐ and lipid‐rich animal‐derived foods are generally more prone to AGE accumulation during cooking, particularly when exposed to dry heat [39]. Grilling, roasting, broiling, baking, and frying promote AGE formation more strongly than moist‐heat methods such as boiling, steaming, stewing, or poaching, largely because lower water activity and higher surface temperatures accelerate dehydration, oxidation, and carbonyl generation [40, 41]. Recent food‐matrix analyses have moved beyond single‐marker measurement toward broader AGE profiling across dairy, meat, and plant‐protein matrices [42, 43]. These studies reinforce that CML, CEL, MG‐H1, pyrraline, and fluorescent AGEs capture different aspects of dietary AGE exposure rather than serving as interchangeable markers [44]. Controlled feeding studies further support cooking method as a modifiable determinant of systemic AGE exposure, although food AGE content should not be treated as a direct surrogate of tissue AGE burden [45].

Smoking represents another major exogenous source of AGE‐related stress. Tobacco smoke contains reactive glycation products and carbonyl compounds capable of modifying circulating and structural proteins, and smokers have been reported to show higher AGE‐modified apolipoprotein B and circulating AGE levels than nonsmokers [13, 46, 47]. More recent work also indicates that smoking acutely alters the RAGE axis: in intermittent smokers, smoking three cigarettes within 1 h rapidly reduced circulating soluble RAGE levels, an effect associated with neutrophil activation and increased CD11b expression [48]. These findings suggest that environmental AGE exposure should also be considered as a source of reactive carbonyl stress and receptor‐axis perturbation. Environmental pollutants may represent a third, less well‐quantified source of AGE‐related carbonyl stress. Atmospheric chemistry studies show that glyoxal and methylglyoxal are reactive dicarbonyls generated from volatile organic compound oxidation and can participate in secondary organic aerosol formation [49]. Direct analytical studies have detected glyoxal and methylglyoxal in fine particulate matter (PM2.5), and biomass‐burning plumes can contain glyoxal, methylglyoxal, and related carbonyl compounds [50, 51]. Although these atmospheric dicarbonyls should not be equated directly with tissue AGE accumulation, inhaled particulate and combustion‐derived pollutants may increase oxidative and inflammatory stress and may perturb the AGE/RAGE axis. For example, diesel particulate matter upregulated RAGE expression in pulmonary epithelial cells and promoted inflammatory signaling through nuclear factor kappa B (NF‐κB) [52]. Taken together, dietary AGEs, tobacco smoke, and pollutant‐derived dicarbonyls represent distinct exogenous inputs into AGE‐related carbonyl and receptor‐axis stress. Nevertheless, their systemic contribution remains difficult to quantify. Dietary AGEs undergo digestion, microbial metabolism, epithelial interaction, absorption, renal filtration, and fecal or urinary excretion, while free and protein‐bound AGEs may have substantially different bioavailability. Emerging evidence further indicates that colonic microbiota can metabolize specific diet‐derived glycation products: human fecal communities and *Cloacibacillus evryensis* degrade CML under anaerobic conditions, whereas *Collinsella intestinalis* expresses an inducible pathway for the metabolism of fructoselysine, an Amadori product and AGE precursor [53, 54]. The detection of putative microbial CML metabolites in human urine supports the in vivo relevance of these processes and suggests that interindividual differences in microbiota composition could modify the chemical forms available for absorption and excretion [55]. Nevertheless, direct evidence that microbiota composition materially determines systemic or tissue AGE burden in humans remains limited. Consequently, exogenous AGE or carbonyl exposure does not directly translate into tissue AGE burden. This uncertainty supports integrating exogenous exposure into the broader AGE burden model, rather than treating dietary intake, smoking, or environmental pollutant exposure as isolated determinants of disease risk.

### 2.3 Detoxification and Clearance of AGEs

AGE accumulation depends not only on formation and exposure, but also on the efficiency of dicarbonyl detoxification, protein quality control, tissue turnover, and renal clearance. The glyoxalase system is the principal enzymatic defense against methylglyoxal‐mediated glycation. In this glutathione‐dependent pathway, glyoxalase 1 (GLO1) catalyzes the conversion of the methylglyoxal–glutathione hemithioacetal to S‐d‐lactoylglutathione, and glyoxalase 2 (GLO2) hydrolyzes this intermediate to d‐lactate while regenerating reduced glutathione [56]. Because methylglyoxal is continuously generated during glycolysis, glyoxalase activity functions as a constitutive antiglycation barrier. Experimental evidence supports the biological importance of this pathway: *Glo1* overexpression reduces hyperglycemia‐induced carbonyl stress, AGE formation, and oxidative injury, whereas impaired glyoxalase activity increases methylglyoxal‐derived protein and nucleotide damage [57, 58]. Recent work has further positioned methylglyoxal–glyoxalase biology as a metabolic stress pathway relevant to insulin resistance and diabetic vascular complications, shifting attention from nonspecific antioxidant protection toward dicarbonyl‐specific detoxification [59]. *Glo1* expression is further coordinated with cellular stress‐response programs, as nuclear factor erythroid 2‐related factor 2 (NRF2) directly promotes GLO1 transcription through an antioxidant response element, linking methylglyoxal detoxification with redox homeostasis and electrophile defense [60]. In parallel, antioxidant defense and proteostasis influence AGE handling indirectly. Oxidative stress accelerates glycoxidation and lipid peroxidation‐derived carbonyl generation, while glutathione availability supports glyoxalase flux [61, 62]. Mild or early protein glycation may be managed through chaperone systems, autophagy, lysosomal degradation, and the ubiquitin–proteasome system, whereas heavily glycated or crosslinked proteins become more resistant to proteolysis and may accumulate in long‐lived tissues [63]. Thus, glutathione depletion, impaired autophagy, proteasomal dysfunction, and oxidative stress can increase AGE burden even without a major increase in glucose exposure.

Renal clearance is a second major determinant of systemic AGE burden. Proteolysis of AGE‐modified proteins generates AGE peptides and free AGE adducts, which enter the circulation and are cleared largely through glomerular filtration, tubular handling, and urinary excretion [64]. Loss of kidney function therefore increases circulating AGE levels by reducing clearance, while uremia, inflammation, oxidative stress, and metabolic dysregulation simultaneously promote further AGE formation [65]. Clinical studies strengthen this clearance‐based interpretation. In Type 2 diabetes, a serum composite score based on five free AGE adducts predicted renal function loss, macroalbuminuria, and high‐risk chronic kidney disease (CKD), supporting the use of chemically defined free adducts as risk‐linked biomarkers [66]. A study using automated preanalytical processing for serum AGE measurement in patients with Type 2 diabetes, where higher serum AGEs were associated with progressive vascular complications, suggesting that improved analytical workflows may make AGE profiling more scalable for clinical studies [67]. Kidney‐focused reviews similarly emphasize that AGE peptides and free adducts accumulate as glomerular filtration declines, making renal dysfunction both a driver and consequence of systemic AGE stress [68, 69]. Clinically, circulating or urinary AGE adducts should therefore be interpreted as composite signals that integrate formation, proteolytic release, filtration, tubular handling, and tissue turnover. This distinction is essential for studies that aim to modify AGE biology in vivo rather than merely inhibit glycation chemistry in vitro. Together, these processes define AGE burden as a lifecycle shaped by formation, exposure, detoxification, clearance, and tissue accumulation, as summarized in Figure 1.

**FIGURE 1:**
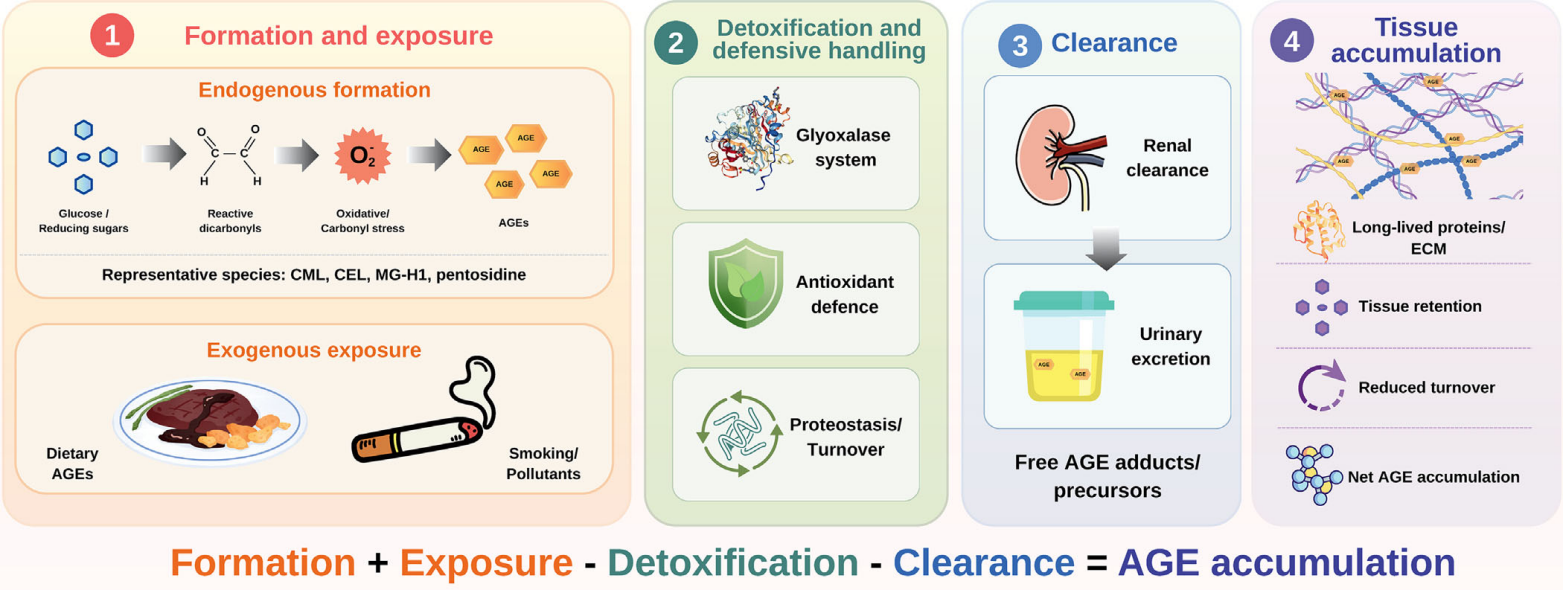
AGE lifecycle: formation, detoxification, clearance, and accumulation. Schematic overview of the major determinants of AGE burden. Endogenous AGE formation is driven by reducing sugars, reactive dicarbonyls, oxidative/carbonyl stress, and subsequent generation of AGE species such as CML, CEL, MG‐H1, and pentosidine. Exogenous exposure arises mainly from dietary AGEs, smoking, and environmental pollutants. AGE burden is counterbalanced by detoxification and defensive handling, including the glyoxalase system, antioxidant defense, proteostasis, and protein turnover, as well as renal clearance and urinary excretion of AGE adducts and precursors. When formation and exposure exceed detoxification and clearance, AGEs accumulate in long‐lived proteins and extracellular matrix, leading to tissue retention, reduced turnover, and net AGE accumulation. *Abbreviations*: AGE, advanced glycation end product; CEL, Nε‐carboxyethyllysine; CML, Nε‐carboxymethyllysine; ECM, extracellular matrix; MG‐H1, methylglyoxal‐derived hydroimidazolone.

### 2.4 AGE Receptors and Signaling Entry Points

Although AGE‐mediated tissue injury can arise directly from chemical modification of proteins and extracellular matrix components, receptor‐mediated recognition provides an additional route through which AGEs are translated into cellular responses. RAGE, encoded by AGER, is the best‐characterized AGE‐binding receptor and belongs to the immunoglobulin superfamily [70, 71]. Its extracellular region contains one V‐type and two C‐type immunoglobulin‐like domains that recognize AGEs and several other damage‐associated ligands, including S100/calgranulins, HMGB1, amyloid‐β, and nucleic acid‐derived ligands, whereas its short cytoplasmic tail is required for intracellular signal transduction [72, 73]. RAGE should therefore be regarded not simply as an AGE receptor, but as a stress‐responsive pattern‐recognition receptor that links glycation stress with inflammatory and tissue‐damage signaling [74]. A conceptual advance is the identification of DIAPH1 as a key intracellular effector of RAGE signaling. The cytoplasmic domain of RAGE interacts with DIAPH1, and pharmacological disruption of this interface reduced diabetic complications in experimental models [19]. This intracellular interaction has attracted attention as a potentially more selective therapeutic target than broad extracellular blockade of RAGE ligands, particularly in vascular and cardiometabolic disease contexts [75]. Thus, AGE pathogenicity is shaped not only by ligand formation, but also by receptor expression, ligand availability, cellular context, and downstream signaling capacity, which collectively determine whether glycated proteins are cleared, contained, or amplified into tissue injury.

Soluble RAGE (sRAGE) further complicates the interpretation of AGE–RAGE biology. Circulating sRAGE consists mainly of cleaved RAGE generated by proteolytic shedding of membrane‐bound RAGE and endogenous secretory RAGE generated through alternative splicing; both forms lack the transmembrane and cytoplasmic domains required for intracellular signaling [76, 77, 78]. Functionally, sRAGE may limit ligand access to membrane‐bound RAGE by binding RAGE ligands without initiating intracellular signaling, which has led to its common description as a decoy receptor [79]. However, this interpretation is context dependent: circulating sRAGE concentrations may reflect ligand sequestration, receptor shedding, inflammatory activity, renal function, tissue injury, or disease‐associated receptor turnover. In Type 2 diabetes, circulating AGEs, sRAGE isoforms, and AGE/sRAGE ratios have been associated with mortality and cardiovascular complications, suggesting that they may serve as integrated biomarkers of AGE–RAGE activity, but should not be interpreted in isolation [80]. Other AGE‐binding systems, including AGE‐R1/OST‐48, AGE‐R2/80K‐H, AGE‐R3/Galectin‐3, and scavenger receptors such as CD36, SR‐A, and LOX‐1, may contribute to AGE binding, endocytosis, degradation, or inflammatory modulation [81, 82, 83]. These receptors are less central than RAGE in canonical proinflammatory AGE signaling but may contribute to AGE handling and cellular clearance. Together, these receptor systems bridge AGE accumulation with cellular fate decisions, determining whether AGE‐modified ligands are cleared, tolerated, or converted into chronic stress signals; this provides the biological entry point for the receptor‐independent and receptor‐dependent mechanisms of tissue injury discussed in the next section.

## 3 Pathogenesis of AGE‐Mediated Tissue Injury

AGE‐mediated tissue injury occurs through several overlapping but mechanistically distinct routes. Receptor‐independent damage arises from direct modification of proteins and extracellular matrix, leading to altered protein function, impaired proteostasis, matrix crosslinking, and abnormal tissue mechanics. Receptor‐dependent injury is mediated mainly through AGE–RAGE signaling and downstream redox‐inflammatory amplification. These two routes converge on recurrent pathogenic programs, including oxidative stress, mitochondrial dysfunction, immune dysregulation, cellular senescence, vascular barrier injury, fibrosis, and feed‐forward loops that sustain chronic tissue damage. This section therefore separates the mechanisms analytically while emphasizing that they usually operate together in disease tissues.

### 3.1 Receptor‐Independent Mechanisms

Receptor‐independent AGE toxicity arises from direct chemical modification of macromolecules and therefore does not require RAGE activation. Glycation of lysine, arginine, and other nucleophilic residues alters local charge, steric configuration, hydrogen bonding, hydrophobicity, and susceptibility to enzymatic cleavage, thereby impairing protein folding, catalytic activity, ligand binding, and structural stability [84, 85]. These effects are particularly relevant for long‐lived proteins, in which even slow glycation reactions can accumulate over years. Lens crystallins provide a classical example, as glycation reduces solubility and chaperone‐like function, promoting higher‐molecular‐weight aggregation and loss of optical transparency [86]. More broadly, glycated proteins can impose a proteostatic burden by becoming less amenable to normal degradation. An in vitro study in pancreatic ductal epithelial cells showed that intracellular protein AGEs increased aggregation and protease resistance, reduced global ubiquitination and multiple E3 ligases, and shifted protein clearance toward autophagy, indicating that AGE accumulation can directly disrupt ubiquitin–proteasome and compensatory autophagic pathways [87]. Although the disease context differs from diabetic complications, this study illustrates a mechanistic principle: AGEs can damage the protein quality‐control system itself, thereby converting glycation from a chemical modification into a self‐reinforcing proteostatic stress.

A second major receptor‐independent mechanism is covalent crosslinking of extracellular matrix proteins. Collagen and elastin are especially susceptible because of their abundance and long half‐lives in vascular walls, basement membranes, myocardium, kidney, tendon, skin, and other mechanically active tissues [88]. AGE‐derived crosslinks restrict collagen molecular sliding, reduce matrix degradability, alter hydration and fibrillar architecture, and impair tissue capacity to dissipate mechanical stress [89]. These effects extend beyond increased matrix stiffness, as AGE‐modified matrices may also alter viscoelastic behavior, stress relaxation, cellular traction, and downstream mechanotransductive signaling [90]. A study in precirrhotic liver tissue showed that glycated proteins increased matrix viscoelasticity and promoted hepatocellular carcinoma progression through integrin‐β1–tensin‐1–YAP mechanosignaling, while inhibition of AGE formation, restoration of AGE clearance, or disruption of AGE crosslinks reduced matrix viscoelasticity and tumor growth [91]. These findings broaden receptor‐independent AGE biology beyond a simple stiffness model and support the view that AGE‐modified matrices can regulate cell behavior through altered mechanics, degradation resistance, and cell–matrix signaling. This separation has therapeutic relevance: RAGE blockade may attenuate inflammatory amplification, but it is unlikely to reverse established protein dysfunction, proteostatic failure, or matrix crosslinking. Receptor‐independent pathology therefore supports early prevention of AGE formation, improved proteostatic resilience, and crosslink‐directed strategies as mechanistically distinct from downstream anti‐inflammatory interventions.

### 3.2 AGE–RAGE Signaling Axis

The AGE–RAGE axis represents the principal receptor‐dependent mechanism by which AGEs amplify cellular stress responses. RAGE recognizes multiple AGE‐modified ligands, including CML‐modified proteins, and ligand engagement initiates intracellular signaling rather than serving primarily as a clearance mechanism. RAGE activation promotes interconnected downstream pathways, including generation of reactive oxygen species (ROS) through NADPH oxidase, activation of mitogen‐activated protein kinases, NF‐κB signaling, and transcriptional induction of inflammatory cytokines, chemokines, adhesion molecules, and other stress‐responsive mediators [92, 93]. The magnitude and consequence of these responses are highly context dependent, reflecting differences in ligand composition, receptor abundance, redox status, metabolic stress, and cell‐specific signaling architecture [94]. In endothelial cells, monocytes/macrophages, smooth muscle cells, podocytes, neurons, and epithelial cells, AGE–RAGE signaling can therefore produce distinct but overlapping outputs, including endothelial activation, leukocyte recruitment, oxidative stress, impaired reparative responses, and inflammatory sensitization [95, 96, 97]. Signal persistence is a defining feature of RAGE biology. AGE‐induced NF‐κB activation can upregulate RAGE expression, thereby forming a feed‐forward circuit in which sustained ligand exposure reinforces receptor abundance and prolongs inflammatory signaling [98]. This mechanism is particularly relevant in AGE‐enriched tissues, where persistent ligand availability may maintain cellular activation despite fluctuations in the initial metabolic trigger.

Recent studies have refined the AGE–RAGE paradigm by emphasizing intracellular signal propagation together with extracellular ligand recognition. The cytoplasmic tail of RAGE is essential for signaling and interacts with DIAPH1, a formin‐family actin regulatory protein that links RAGE activation to cytoskeletal remodeling, inflammatory gene expression, and cell migration [19, 99]. This RAGE–DIAPH1 interface has emerged as a selective signaling node, potentially offering a more precise therapeutic strategy than global inhibition of RAGE ligand binding, given the multiligand nature of RAGE and the context‐dependent functions of its ligands [100]. In 2025, structural and functional studies showed that the small‐molecule antagonist RAGE406R inhibits RAGE‐mediated DIAPH1 activation, suppresses actin polymerization and inflammatory responses in cellular models, reduces CCL2 expression in macrophages derived from individuals with Type 1 diabetes, and improves inflammatory and wound‐healing outcomes in mouse models [20]. Although these findings remain preclinical, they support a more nuanced interpretation of AGE–RAGE signaling as an amplification axis with druggable intracellular checkpoints. Within the framework of this review, receptor‐independent AGE damage accounts for direct protein and matrix dysfunction, whereas AGE–RAGE signaling explains how AGE accumulation is converted into sustained oxidative, inflammatory, vascular, and immune activation.

### 3.3 Convergent Pathogenic Programs

AGE‐mediated injury converges on several recurring tissue programs. These programs are not independent pathways; oxidative stress, inflammation, senescence, barrier dysfunction, and fibrosis often reinforce one another within the same tissue. They are separated here to clarify dominant mechanisms and disease‐specific interpretation.

#### 3.3.1 Oxidative Stress and Mitochondrial Dysfunction

Oxidative stress represents a central downstream consequence of AGE burden, arising from both receptor‐mediated signaling and receptor‐independent glycoxidative chemistry. Under pathological AGE burden, however, redox signaling becomes maladaptive. AGE–RAGE‐induced cytosolic ROS can promote mitochondrial permeability transition, impair respiratory complex function, and increase mitochondrial superoxide generation, particularly in hyperglycemic environments where reducing substrates remain abundant [101, 102, 103]. Mitochondrial injury then becomes both a consequence and amplifier of AGE stress, because impaired oxidative phosphorylation, altered mitochondrial membrane potential, defective mitophagy, and excess mitochondrial ROS can further increase oxidative damage and dicarbonyl generation. Recent work has added another layer to this model by showing that AGEs can induce mitochondrial damage in endothelial cells and promote migrasome‐associated mitocytosis, suggesting that AGE‐induced mitochondrial stress may influence intercellular communication and endothelial injury beyond conventional ROS signaling [104]. Thus, oxidative stress and mitochondrial dysfunction form a self‐reinforcing axis that links AGE accumulation to vascular, renal, neural, and metabolic tissue injury.

#### 3.3.2 Inflammation and Immune Dysregulation

Inflammation is another convergent output of AGE burden, reflecting receptor‐mediated immune activation as well as secondary responses to damaged proteins, altered matrices, and stressed cells. AGEs influence immune responses according to ligand composition, receptor availability, cell lineage, metabolic state, tissue microenvironment, and coexisting danger signals. In macrophages, AGEs can promote a proinflammatory phenotype characterized by increased expression of IL‐6, TNF‐α, inducible nitric oxide synthase, CD11c, and CD86 [105]. Tissue‐specific evidence supports the relevance of this mechanism in metabolic inflammation: in nonalcoholic steatohepatitis, RAGE expression was increased in recruited macrophages, and macrophage RAGE activation promoted inflammatory responses in human tissue and experimental models [106]. AGE‐driven immune dysregulation should not be reduced to a uniform proinflammatory effect. In some settings, AGE accumulation may prime cytokine release and inflammasome‐associated pathways; in others, chronic exposure may impair innate immune responsiveness or resolution [107]. This context dependence is important for later disease sections, because the inflammatory output of AGE biology differs between diabetic wounds, kidney disease, atherosclerosis, neurodegeneration, and fibrotic tissues. The common theme is not a single immune phenotype, but persistent distortion of immune activation, resolution, and repair.

#### 3.3.3 Cellular Senescence and Maladaptive Stress Responses

AGE accumulation can promote cellular senescence by imposing persistent oxidative, proteostatic, mitochondrial, and inflammatory stress. Senescent cells are characterized by stable cell‐cycle arrest, altered metabolism, resistance to apoptosis, and secretion of senescence‐associated factors that can modify neighboring cells and tissue microenvironments [108, 109]. In AGE‐rich settings, this process may be driven by DNA damage, mitochondrial dysfunction, impaired mitophagy, protein aggregation, and chronic receptor‐mediated stress signaling. Therefore, AGE‐induced senescence should also be interpreted as an active pathogenic program that reduces regenerative capacity, impairs tissue repair, and sustains inflammatory and fibrotic signaling through the senescence‐associated secretory phenotype. In bone marrow mesenchymal stem cells, AGE accumulation aggravated senescence, promoted mitochondrial dysfunction, and inhibited mitophagy through a SIRT3‐dependent mechanism, while SIRT3 restoration alleviated senescence and age‐related bone loss in experimental models. In endothelial cells, AGEs have also been linked to senescence‐associated loss of vascular resilience, suggesting that senescence may contribute to barrier dysfunction and impaired repair [110]. These findings suggest that AGE‐associated senescence integrates mitochondrial quality control, cytoskeletal regulation, barrier function, and tissue repair. Within chronic metabolic disease, such maladaptive stress responses may help explain why AGE accumulation is associated with impaired wound healing, vascular aging, renal dysfunction, and degenerative tissue remodeling.

#### 3.3.4 Vascular Dysfunction and Barrier Injury

The vasculature is a major target of AGE‐mediated injury because endothelial cells are continuously exposed to circulating AGE adducts, inflammatory mediators, oxidative stress, and mechanical forces. AGE accumulation promotes endothelial dysfunction through reduced nitric oxide bioavailability, increased oxidative stress, adhesion molecule induction, cytoskeletal remodeling, and disruption of intercellular junctions [111]. These changes impair vasodilation, increase leukocyte adhesion, and compromise vascular barrier integrity. Barrier injury is particularly consequential in microvascular beds where increased permeability permits plasma protein leakage, tissue edema, inflammatory cell recruitment, and impaired perfusion [112]. Mechanistically, AGE exposure has been shown to disrupt VE‐cadherin‐associated junctional complexes and increase endothelial permeability, while more recent studies have identified additional pathways involving β‐catenin phosphorylation, ADAM10 upregulation, mitochondrial oxidative stress, and miR‐1‐3p/MLCK signaling [113]. These mechanisms are relevant across multiple barriers, including the blood–retinal barrier, blood–brain barrier, glomerular filtration barrier, pulmonary endothelial barrier, and intestinal vascular interface, although the dominant cell types and junctional structures differ by tissue [114]. The key concept is that AGE‐mediated vascular injury is not limited to endothelial activation but also involves structural and functional failure of barrier systems that regulate tissue homeostasis. This provides a mechanistic bridge to diabetic microangiopathy, neurovascular dysfunction, kidney injury, and chronic inflammatory tissue damage, which are discussed in later disease‐specific sections.

#### 3.3.5 Extracellular Matrix Remodeling and Fibrosis

Extracellular matrix remodeling and fibrosis represent a major tissue‐level consequence of chronic AGE burden. Fibrosis extends beyond AGE‐mediated crosslinking and reflects a broader remodeling program involving fibroblast and myofibroblast activation, excessive matrix deposition, altered matrix metalloproteinase/tissue inhibitor of metalloproteinase balance, reduced degradability, and impaired resolution of repair responses [115]. AGEs can contribute to this process through several converging mechanisms. AGE‐modified matrix is less permissive to normal proteolytic turnover, which can limit macrophage‐ and protease‐mediated matrix resolution and prolong the persistence of damaged extracellular scaffolds. In scarred liver tissue, AGE‐mediated extracellular matrix modification impaired macrophage‐dependent remodeling, supporting a model in which glycation stress contributes to fibrosis by restricting matrix degradation and repair rather than merely increasing matrix stiffness [18]. In renal injury, proximal tubular RAGE has been shown to promote fibrosis through a STAT3/ATG7‐dependent autophagy pathway, linking AGE–RAGE signaling with maladaptive epithelial stress responses and fibrotic remodeling [116]. In the heart, the AGE–RAGE axis contributes to myocardial fibrosis by activating cardiac fibroblasts and autophagy‐related pathways in heart failure models [117]. These findings update the traditional view of AGE‐associated fibrosis as passive matrix accumulation. Instead, AGEs appear to promote a pathological repair niche in which modified extracellular matrix, persistent inflammatory cues, autophagy‐dependent stress responses, epithelial and fibroblast plasticity, and myofibroblast persistence converge. This distinction provides therapeutic insights: inhibiting AGE formation may reduce new matrix damage, whereas established fibrosis may require strategies that restore matrix degradability, interrupt profibrotic signaling, or remodel the AGE‐modified extracellular niche.

### 3.4 Feed‐Forward Amplification Loops

AGE‐mediated tissue injury is sustained by feed‐forward loops in which glycation, oxidative stress, inflammation, and impaired clearance reinforce one another. ROS generated through AGE–RAGE signaling, mitochondrial dysfunction, NADPH oxidase activation, and lipid peroxidation can accelerate the formation of reactive carbonyl intermediates, thereby increasing the generation of new AGEs [59]. In parallel, AGE‐driven inflammatory signaling promotes cytokine release, leukocyte recruitment, endothelial activation, and tissue stress, all of which can intensify oxidative and metabolic imbalance [118]. This bidirectional relationship is particularly relevant in chronic metabolic and cardiorenal disease, where hyperglycemia, inflammation, mitochondrial dysfunction, renal impairment, and reduced proteostatic capacity coexist. Under these conditions, AGEs are not simply final products of prior metabolic injury; they become amplifiers that perpetuate the biochemical environment required for their continued formation. Recent work has extended this model by linking AGE–ROS signaling to ferroptosis‐related pathways in diabetic complications, suggesting that AGE‐driven redox imbalance may also intersect with iron‐dependent lipid peroxidation and regulated cell death [119]. These loops explain why late blockade of a single downstream mediator may be insufficient once carbonyl generation, oxidative stress, inflammation, and impaired clearance have become mutually reinforcing. Therapeutic strategies may therefore need to interrupt AGE biology earlier, or at multiple levels, before irreversible matrix and tissue remodeling dominate. The integrated receptor‐independent, receptor‐dependent, and feed‐forward mechanisms of AGE‐mediated tissue injury are summarized in Figure 2 and provide the mechanistic basis for interpreting AGE relevance across disease domains and clinical biomarkers.

**FIGURE 2:**
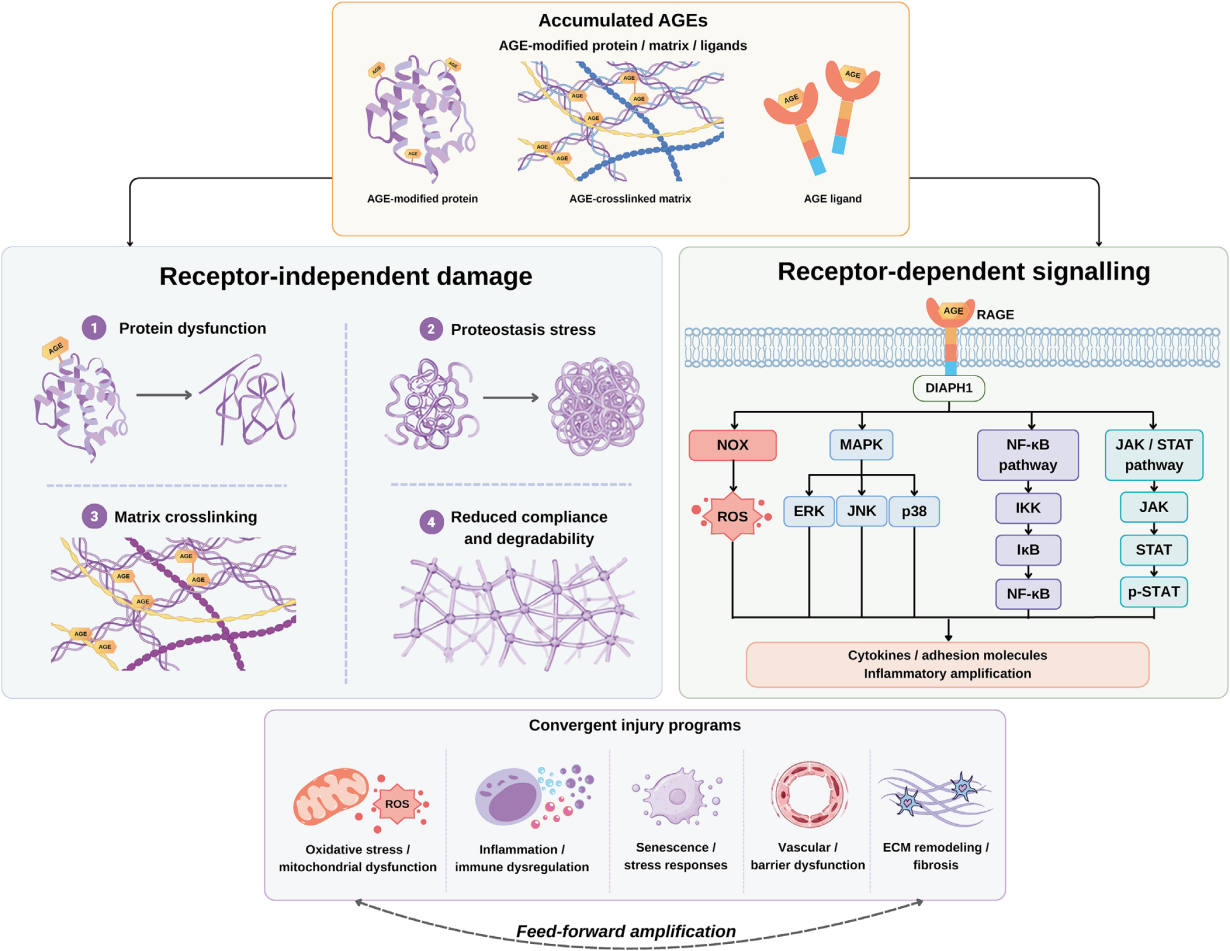
Unified molecular pathogenesis of AGE‐mediated tissue injury. AGE accumulation contributes to tissue injury through receptor‐independent and receptor‐dependent mechanisms. Receptor‐independent damage results from direct modification of proteins and extracellular matrix, leading to protein dysfunction, proteostatic stress, matrix crosslinking, and reduced tissue compliance or degradability. Receptor‐dependent signaling is mediated mainly through AGE–RAGE engagement and downstream DIAPH1‐associated signaling, activating NOX‐derived ROS production, MAPK pathways, NF‐κB signaling, JAK/STAT signaling, cytokine expression, adhesion molecule induction, and inflammatory amplification. These mechanisms converge on recurring injury programs, including oxidative stress and mitochondrial dysfunction, inflammation and immune dysregulation, senescence and maladaptive stress responses, vascular and barrier dysfunction, and extracellular matrix remodeling or fibrosis. Feed‐forward amplification further sustains AGE‐driven tissue injury. *Abbreviations*: AGE, advanced glycation end product; DIAPH1, diaphanous‐related formin 1; ECM, extracellular matrix; JAK, Janus kinase; MAPK, mitogen‐activated protein kinase; NF‐κB, nuclear factor kappa B; NOX, NADPH oxidase; RAGE, receptor for advanced glycation end products; ROS, reactive oxygen species; STAT, signal transducer and activator of transcription.

## 4 Disease Spectrum and Clinical Assessments

The clinical relevance of AGEs differs substantially across diseases because AGE formation, tissue retention, renal clearance, receptor activation, and measurement compartment vary by organ system. This section therefore evaluates AGE biology according to evidence maturity and clinical interpretability. Throughout this section, evidence is distinguished as biochemical or cell‐based, preclinical in vivo, observational human, longitudinal or prognostic human, and interventional clinical evidence. Mechanistic and animal studies establish biological plausibility, while observational human studies support association but remain vulnerable to confounding; stronger causal inference requires convergent longitudinal evidence and, ideally, intervention studies demonstrating target engagement and functional or clinical benefit. This evidence framework guides the comparative discussion of the disease domains that follows.

Diabetes is used as the central clinical anchor because it has the strongest mechanistic and longitudinal evidence linking glycation, dicarbonyl stress, tissue AGE accumulation, and complications. Cardiovascular disease and CKD are then discussed as major settings in which AGE burden intersects with vascular stiffness, tissue remodeling, and impaired clearance. Neurovascular, retinal, fibrotic, and aging‐related tissues are considered more cautiously, because their evidence base often combines mechanistic plausibility, preclinical models, and associative human data. The section concludes by clarifying how AGE biomarkers should be interpreted across biological compartments.

### 4.1 Diabetes

Diabetes is the clearest clinical setting in which accelerated AGE formation, tissue retention, and measurable complications intersect. At the clinical level, its relevance lies in the sustained metabolic environment created by chronic hyperglycemia, increased glycolytic flux, oxidative stress, and reduced stress‐buffering capacity. In this setting, methylglyoxal‐driven dicarbonyl stress provides a rapid route to lysine‐ and arginine‐derived AGE adducts, while glyoxalase activity, proteostasis, tissue protein turnover, and renal handling determine whether these modifications remain transient or accumulate as clinically relevant AGE burden [8, 59]. Recent adductomic studies strengthen this view by showing that methylglyoxal‐derived adducts can function as prognostic markers of diabetic kidney disease in Type 1 diabetes, supporting a shift from nonspecific “total AGE” measurements toward species‐defined risk markers [10].

A clinically distinctive feature of diabetic AGE biology is the durability of glycation‐derived tissue injury. Long‐lived collagen, elastin, basement membrane proteins, and other extracellular matrix components can retain AGE modifications over prolonged periods, thereby preserving a biochemical record of prior metabolic stress. This persistence may contribute to metabolic memory, in which earlier glycemic exposure continues to influence later complication risk despite subsequent improvement in glycemic control [120]. Skin collagen analyses from the Diabetes Control and Complications Trial/Epidemiology of Diabetes Interventions and Complications cohort support this interpretation, as glucosepane and MG‐H1 were independently associated with long‐term microvascular complication progression in Type 1 diabetes [121, 122]. These data support AGE‐linked tissue memory, while recognizing that metabolic memory also involves epigenetic, inflammatory, vascular, and mitochondrial mechanisms [120, 123]. Diabetes should therefore be treated as the central clinical anchor for accelerated AGE generation and tissue retention, whereas organ‐specific consequences are considered in the following cardiovascular, renal, neural, retinal, and aging‐related subsections. The key interpretive point is that diabetic AGE burden is not a single measurement: circulating free adducts, protein‐bound adducts, tissue crosslinks, skin autofluorescence, and urinary markers capture different combinations of formation, retention, turnover, and clearance. These findings provide longitudinal and prognostic human evidence but do not establish that lowering a specific AGE species will reduce complication risk.

### 4.2 Cardiovascular Disease

AGE‐related mechanisms are implicated in cardiovascular disease through vascular wall modification, endothelial stress, lipoprotein alteration, and myocardial extracellular matrix remodeling. In the arterial wall, AGE‐modified collagen and elastin reduce matrix compliance and increase resistance to enzymatic turnover, contributing to arterial stiffening and adverse vascular remodeling [89]. AGE‐modified lipoproteins may further aggravate vascular injury by altering lipoprotein recognition, macrophage uptake, and endothelial handling [124]. These mechanisms are clinically relevant to hypertension, arterial stiffness, atherosclerosis, diabetic macrovascular disease, and heart failure, but their relative contribution varies by glycemic status, renal function, aging, inflammation, and lipid burden [125]. Human studies support this association at the biomarker level [126]. Skin autofluorescence, a noninvasive surrogate of tissue AGE accumulation, has been associated with vascular stiffness across normoglycemic, prediabetic, and diabetic individuals [127], stable coronary artery disease [128], subclinical cardiovascular disease in the Rotterdam Study [129], and imaging‐defined subclinical atherosclerosis in coronary and carotid arteries [130]. These findings suggest that tissue AGE accumulation may capture cumulative vascular injury beyond short‐term glucose or lipid measurements, although skin autofluorescence remains a compartment‐specific surrogate rather than a direct measure of individual AGE species.

Recent experimental work has strengthened the mechanistic plausibility of a causal link between glycation stress and vascular stiffening, although the human evidence discussed above remains observational. In a 2025 study, chronic methylglyoxal exposure increased pulse‐wave velocity and aortic stiffness in mice, while ex vivo exposure of mouse aortic rings to methylglyoxal or MG‐H1 increased elastic modulus. The study further implicated oxidative stress, endothelial senescence, reduced nitric oxide bioavailability, and glyoxalase‐related detoxification in methylglyoxal‐driven aortic stiffening [131]. The value of this study lies in connecting a chemically defined dicarbonyl stressor with a functional vascular endpoint, rather than relying solely on cross‐sectional associations between bulk AGE signals and cardiovascular risk. In the myocardium, AGE‐modified matrix and AGE–RAGE‐associated fibroblast activation may contribute to myocardial stiffness and fibrotic remodeling, with experimental heart failure studies supporting involvement of the AGE–RAGE axis in cardiac fibroblast activation [117]. Cardiovascular AGE biology should therefore be interpreted as a convergence of tissue‐retained matrix modification, dicarbonyl stress, vascular inflammation, endothelial dysfunction, and myocardial remodeling. Clinical translation will depend on whether AGE measurements can improve risk stratification, identify AGE‐active vascular phenotypes, or provide target‐engagement readouts for interventions aimed at dicarbonyl detoxification, matrix remodeling, or RAGE‐axis inhibition.

### 4.3 Chronic Kidney Disease

The kidney occupies a dual position in AGE biology: it is a major route for AGE elimination and a target of AGE‐mediated injury. Proteolysis of AGE‐modified proteins generates AGE peptides and free adducts that enter the circulation and are cleared through glomerular filtration, tubular handling, and urinary excretion [132]. When renal function declines, circulating AGE adducts rise because clearance is reduced. In advanced renal failure, plasma glycation free adducts are markedly increased in patients receiving peritoneal dialysis or hemodialysis, supporting the role of renal excretion in systemic AGE removal [133]. Fractional excretion studies in Type 1 diabetes further suggest that altered tubular handling of glycation adducts may identify early renal functional decline before advanced loss of filtration capacity [134]. Thus, AGE concentrations in kidney disease cannot be interpreted simply as markers of formation. They may reflect increased dicarbonyl stress, proteolytic release from modified tissues, reduced glomerular filtration, altered tubular processing, dietary exposure, or dialysis‐related clearance.

AGE accumulation may also contribute directly to renal pathology. In diabetic kidney disease, hyperglycemia and dicarbonyl stress promote AGE formation in glomerular, vascular, and tubulointerstitial compartments, where AGE‐modified matrix, podocyte stress, endothelial dysfunction, mesangial expansion, tubular injury, and impaired matrix turnover can participate in albuminuria and progressive loss of filtration capacity [135]. In Type 2 diabetes, a serum composite score based on chemically defined free AGE adducts predicted renal function loss, macroalbuminuria, and high‐risk CKD [66]. In Type 1 diabetes, serum protein glycation products, including AGEs and MG‐H1, were associated with kidney disease progression and incident major adverse cardiovascular events in the FinnDiane cohort [136]. Experimental evidence also links RAGE activation to fibrotic renal remodeling, as proximal tubular RAGE promoted fibrosis through a STAT3/ATG7‐dependent autophagy pathway in obstructive nephropathy [116], and methylglyoxal‐derived AGE exposure aggravated renal lesions in diabetic *db/db* mice through oxidative, mitochondrial, inflammatory, and inflammasome‐linked pathways [137]. The human studies therefore provide prognostic or clearance‐related associations, whereas the pathway‐level causal evidence derives mainly from experimental models. Kidney disease therefore creates a self‐reinforcing AGE context in which impaired clearance raises systemic AGE exposure, while local AGE accumulation promotes renal inflammation, matrix injury, and fibrosis. Clinically, AGE biomarkers in CKD should be interpreted alongside estimated glomerular filtration rate, albuminuria, tubular function, dialysis status, inflammation, and nutritional exposure.

### 4.4 Neurovascular, Retinal, and Aging‐Related Tissue Dysfunction

AGEs are relevant to nervous system and retinal disease because neural tissues depend on long‐lived proteins, intact microvascular barriers, glial support, mitochondrial homeostasis, and efficient proteostasis. In Alzheimer's disease‐related models, AGE–RAGE signaling has been linked to amyloidogenic amyloid precursor protein processing and tau phosphorylation through cathepsin B and asparagine endopeptidase pathways, providing a molecular connection between glycation stress and classical neurodegenerative pathology [138]. Recent reviews further support an AGE/RAGE–oxidative stress framework in late‐onset Alzheimer's disease and neuroinflammatory disorders, while emphasizing that human causality remains difficult to separate from aging, diabetes, vascular disease, renal dysfunction, and chronic inflammation [139, 140]. Peripheral nerves provide a clinically relevant diabetic model. In Type 2 diabetes, intradermal AGE deposition was associated with reduced sciatic nerve structural integrity and arterial stiffness, suggesting that tissue glycation may link vascular injury with peripheral nerve damage [141, 142]. The retina provides a related neurovascular model. AGE accumulation has been associated with diabetic retinal neurovascular‐unit impairment, involving endothelial cells, pericytes, Müller glia, neurons, microglia, and retinal pigment epithelial cells [143, 144, 145]. This is clinically relevant because diabetic retinal disease is now increasingly viewed as an early neurovascular disorder rather than a purely microvascular endpoint.

Compared with diabetes, kidney disease, and cardiovascular disease, evidence in neurodegenerative and aging‐related tissues is more heterogeneous and often relies on associative or preclinical data. AGE accumulation also contributes to aging‐related dysfunction in tissues with slow protein turnover, including skin, tendon, lens, skeletal muscle, and connective tissues. In these compartments, AGE adducts and crosslinks may reduce elasticity, increase protease resistance, disturb cell–matrix signaling, and impair tissue repair [146]. Skeletal muscle illustrates both the biological plausibility and the translational gap [147]. Observational studies suggest that skin autofluorescence and circulating AGE measures are generally associated with poorer muscle outcomes in adults, but much of the evidence remains cross‐sectional [148]. Experimental work further indicates that AGEs can acutely disturb skeletal muscle proteostasis and intracellular signaling, supporting a potential role in muscle dysfunction [149]. Recent reviews have extended this concept to skeletal–muscle atrophy and exercise‐responsive AGE biology, suggesting that physical activity may modify AGE accumulation or AGE–RAGE signaling in muscle, although direct clinical evidence remains limited [150]. Clinical studies need to distinguish tissue‐retained AGEs, circulating free adducts, skin autofluorescence, renal clearance effects, and disease‐specific cellular vulnerability before assigning pathogenic weight to AGE measurements.

### 4.5 Liver Fibrosis and Matrix‐Rich Tissue Dysfunction

Liver fibrosis is a clinically relevant setting for AGE‐related pathology because the liver integrates dietary and endogenous carbonyl exposure, metabolic inflammation, Kupffer‐cell activation, hepatic stellate cell responses, extracellular matrix deposition, and matrix remodeling within the same disease process. Experimental studies suggest that AGEs can act at several levels of this fibrogenic niche. In human hepatic stellate cells, glyceraldehyde‐derived AGEs increased ROS production, proliferation, MCP‐1 secretion, and fibrogenic gene expression, including α‐smooth muscle actin, transforming growth factor‐β1, and collagen‐related transcripts [151]. In vivo, AGE administration aggravated hepatic fibrosis and oxidative stress in bile duct ligation models, while high dietary AGE exposure worsened steatohepatitis, collagen deposition, α‐smooth muscle actin expression, and inflammatory liver injury in diet‐induced NAFLD/NASH models [152, 153]. Mechanistically, AGEs have been linked to NOX2‐dependent oxidative stress, downregulation of Sirt1/Timp3 signaling, activation of TNF‐α‐converting enzyme activity, and fibrogenic transcriptional responses in NASH models and hepatic stellate cells [154]. Human data remain more limited and primarily associative but support clinical relevance: hepatic AGEs were higher in nonalcoholic steatohepatitis than in simple steatosis or control liver tissue and were associated with higher hepatic transforming growth factor‐β1 and α‐smooth muscle actin, whereas serum AGEs and sRAGE did not clearly distinguish groups [155]. More recent clinical evidence further suggests that dietary AGE exposure may have prognostic relevance in advanced chronic liver disease, as higher dietary AGE intake was associated with increased mortality risk in a prospective cohort of patients with cirrhosis [156]. The RAGE axis should be interpreted carefully in this setting. RAGE expression is increased in activated hepatic stellate cells and fibrotic liver compartments, but direct stimulation of stellate cells with AGE‐BSA or CML‐BSA does not uniformly induce a profibrotic phenotype, suggesting that RAGE expression alone does not establish direct AGE‐driven fibrogenesis [157]. However, recent work indicates that redox‐sensitive HMGB1 isoforms can drive liver fibrosis progression or resolution through RAGE‐dependent effects on hepatic stellate cells [158]. Thus, hepatic AGE/RAGE biology is best viewed as part of a broader fibrogenic injury network rather than as a single linear AGE–RAGE pathway.

The matrix dimension further strengthens the relevance of liver fibrosis to AGE biology. In scarred liver tissue, AGE‐mediated extracellular matrix crosslinking altered collagen architecture and impaired macrophage‐dependent matrix remodeling, supporting a receptor‐independent mechanism by which AGEs stabilize an abnormal fibrotic matrix niche [18]. In the precirrhotic liver, glycated matrix increased viscoelasticity and promoted hepatocellular carcinoma progression through integrin‐β1–tensin‐1–YAP mechanosignaling, while reducing AGE formation or modifying AGE‐linked matrix mechanics attenuated tumor‐promoting matrix behavior [91]. These studies indicate that hepatic AGEs should not be viewed only as circulating biomarkers or inflammatory ligands; they may also alter the physical and remodeling properties of the fibrotic tissue scaffold. Related principles may apply to other matrix‐rich organs, including kidney and heart, where AGE–RAGE‐associated mechanisms have been linked to renal fibrotic remodeling and cardiac fibroblast activation [116, 117], and skeletal muscle, where AGE‐crosslinked decellularized matrix reduced regenerative capacity and increased inflammatory responses in an in vivo volumetric muscle loss model [159]. However, liver fibrosis deserves particular emphasis because it combines metabolic carbonyl stress, inflammatory signaling, stellate‐cell‐driven collagen deposition, impaired macrophage‐mediated remodeling, matrix stiffening or viscoelasticity, and clinically measurable progression toward cirrhosis and hepatocellular carcinoma. Future studies should therefore assess liver‐tissue AGE species, matrix‐bound crosslinks, liver stiffness or viscoelasticity, fibrosis stage, macrophage and stellate‐cell activity, and clinical outcomes, rather than relying solely on serum AGE or sRAGE concentrations.

### 4.6 Biomarkers and Analytical Approaches

Clinical AGE assessment depends on the biological compartment being measured. Circulating free AGE adducts in plasma, serum, or urine often reflect recent formation, dietary absorption, proteolytic release, and renal clearance, whereas protein‐bound AGEs reflect modification of circulating or tissue proteins over longer time scales [3]. Tissue‐retained AGEs, especially those in collagen‐rich matrices, may better capture cumulative glycation history and long‐lived structural modification. Skin autofluorescence provides a noninvasive estimate of fluorescent AGE‐related tissue accumulation and has been validated against skin collagen fluorescence and biopsy‐derived AGE measures [160]. It is frequently used in diabetes, cardiovascular disease, kidney disease, and aging‐related research as a surrogate of cumulative tissue glycation [161]. Its interpretation is constrained by age, skin pigmentation, smoking, renal function, device characteristics, and the fact that nonfluorescent AGEs such as CML and glucosepane are not directly measured by autofluorescence. Recent reference‐value work further supports the need to interpret skin autofluorescence against age‐ and population‐appropriate biological ranges rather than as a generic AGE‐burden score [162, 163].

Analytical approaches differ in specificity, scalability, and biological meaning. LC–MS/MS provides the greatest chemical specificity and can quantify defined AGE species and related dicarbonyl‐derived adducts [164, 165]. Skin autofluorescence and other fluorescence‐based readouts are simpler and scalable, but they mainly capture fluorescent AGE‐related signals and cannot distinguish individual adducts [163, 166]. Immunoassays are suitable for larger studies, yet antibody specificity, calibration differences, matrix effects, and epitope heterogeneity limit comparability across cohorts [167, 168]. Dietary AGE databases and food AGE estimates add another layer of uncertainty because cooking method, matrix composition, digestion, absorption, microbiota metabolism, and renal handling influence systemic exposure [169]. For clinical assessment, the AGE readout should be selected according to the research question [164, 165]. For example, plasma or urinary free AGE adducts are most appropriate for examining recent formation–proteolysis–clearance dynamics or pharmacodynamic responses, but they should not be interpreted as direct measures of long‐term tissue crosslinking, particularly when renal function is impaired [170]. Protein‐bound AGEs are better suited to assessing modification of circulating or tissue proteins over their respective turnover periods, but they cannot identify the affected tissue or be assumed to reflect recent dietary exposure [12]. Skin autofluorescence provides a scalable surrogate of cumulative dermal fluorescent AGE‐related burden, but it neither quantifies individual AGE species nor directly captures nonfluorescent crosslinks such as glucosepane [171]. Methylglyoxal, glyoxal, 3‐deoxyglucosone, and their defined adducts are appropriate for assessing dicarbonyl stress and target engagement by carbonyl‐scavenging or glyoxalase‐directed interventions, but they should not be interpreted as measures of total AGE burden [172]. Dietary AGE estimates are useful for comparing exposure or monitoring dietary interventions, but they do not establish absorbed dose or systemic or tissue AGE accumulation [173]. Direct analysis of collagen or other tissue‐bound AGEs is most relevant to long‐term matrix retention and tissue mechanics, but such measurements are invasive and specific to the sampled tissue [174]. These interpretive constraints provide the basis for evaluating whether AGE‐targeted interventions are mechanism‐matched, appropriately staged, and supported by target‐engagement biomarkers, as discussed in the therapeutic section below. Major AGE‐related analytes, biological compartments, measurement approaches, and interpretive considerations are summarized in Table 1.

**TABLE 1:** AGE‐related analytes and readouts: chemical origin, compartment, measurement, and interpretation.

Analyte/readout	Chemical class	Main form/compartment	Preferred method	Main interpretation	References
Fructosyl‐lysine/Amadori products	Early glycation product	Protein‐bound; plasma proteins/hemoglobin	LC–MS/MS; furosine assay	Early glycation exposure	[1, 2, 3, 26]
MGO	Dicarbonyl precursor	Reactive precursor; intracellular/plasma	Derivatization LC–MS/MS	Dicarbonyl stress	[8, 9, 30, 31, 32, 60]
GO	Dicarbonyl precursor	Reactive precursor; plasma/tissue fluids	Derivatization LC–MS/MS	Glycoxidative carbonyl stress	[25, 28, 33]
3‐DG	Dicarbonyl precursor	Reactive precursor; plasma/food‐derived fraction	Derivatization LC–MS/MS	Amadori‐derived carbonyl stress	[8, 9]
Environmental GO/MGO/pollutant‐related dicarbonyls	Exogenous carbonyl stress	Atmospheric PM2.5; biomass‐burning and combustion‐derived exposure	Air‐sampling carbonyl analysis; LC‐DAD/HPLC/exposure assessment	Potential exogenous carbonyl and RAGE‐axis stress; not direct tissue AGE burden	[49, 50, 51, 52]
CML	Lysine AGE	Free adduct; protein‐bound; dietary/tissue	LC–MS/MS	Glycoxidation marker	[33, 164, 165]
CEL	Lysine AGE	Free adduct; protein‐bound; plasma/urine	LC–MS/MS	MGO‐related lysine modification	[34, 164, 165]
MG‐H1	Arginine AGE	Free adduct; protein‐bound; cellular/tissue proteins	LC–MS/MS	MGO‐related arginine modification	[8, 9, 10, 121, 164, 165]
G‐H1	Arginine AGE	Free adduct; protein‐bound; plasma/tissue proteins	LC–MS/MS	GO‐related arginine modification	[3, 164, 165]
Pentosidine	Fluorescent crosslink	Protein‐bound; collagen/matrix‐associated	HPLC fluorescence; LC–MS/MS	Glycoxidative crosslinking	[3, 164, 165]
Glucosepane	Nonfluorescent crosslink	Matrix‐bound collagen; long‐lived tissues	Specialized LC–MS/MS; antiglucosepane detection	Long‐lived collagen crosslinking	[35, 121, 198]
Pyrraline	Dietary/lysine AGE	Food‐derived; peptide‐bound/free adduct	LC–MS/MS; HPLC	Thermal‐processing AGE exposure	[38, 40, 44, 168]
Protein‐bound total AGEs	Composite AGE readout	Serum proteins; tissue proteins; bulk AGE signal	Immunoassay; fluorescence; LC–MS/MS validation	Overall protein AGE signal	[3, 164, 165, 167, 168]
Skin autofluorescence	Composite tissue signal	Skin collagen/dermal fluorophores	Skin autofluorescence device	Long‐term tissue fluorescence	[160, 161, 162, 163, 166]

*Abbreviations*: 3‐DG, 3‐deoxyglucosone; AGE, advanced glycation end product; CEL, Nε‐carboxyethyllysine; CML, Nε‐carboxymethyllysine; G‐H1, glyoxal‐derived hydroimidazolone; GO, glyoxal; HPLC, high‐performance liquid chromatography; LC–MS/MS, liquid chromatography–tandem mass spectrometry; MG‐H1, methylglyoxal‐derived hydroimidazolone; MGO, methylglyoxal.

### 4.7 Translational Barriers in AGE Measurement and Interpretation

The interpretive problem in clinical AGE research is attribution. The same AGE signal can arise from increased endogenous formation, higher exogenous exposure, proteolytic release from modified tissues, reduced filtration, altered tubular handling, inflammatory tissue injury, or assay‐related variation. Clinical reports should therefore specify the sample type, AGE fraction, analytical platform, calibration strategy, renal function, dietary context, and intended biological interpretation. This is especially relevant for skin autofluorescence, which is useful as a noninvasive tissue readout but must be interpreted against age‐ and population‐adjusted reference values rather than as a universal AGE‐burden score [162].

A second barrier is the distinction between association and causation. Human studies show that AGE‐related markers associate with vascular stiffness, kidney disease progression, neuropathy, cardiovascular outcomes, or aging‐related dysfunction, but these associations are frequently confounded by glycemic history, renal function, inflammation, adiposity, smoking, medication use, and comorbid disease [66, 129, 175]. AGE measurements may therefore serve as integrated risk markers without proving that AGE modification is the dominant driver of tissue injury. Stronger translational evidence will require longitudinal studies that connect changes in defined AGE species or tissue AGE readouts with clinically meaningful outcomes, rather than relying only on cross‐sectional associations. Intervention studies should also include target‐engagement biomarkers matched to the proposed mechanism: reduced dicarbonyl adduct formation for dicarbonyl scavengers or glyoxalase‐directed approaches, altered tissue AGE accumulation for long‐term AGE‐lowering strategies, improved AGE clearance for renal or proteostatic interventions, and pathway‐level readouts for RAGE‐directed therapies. The clinical disease domains, measurement compartments, and major determinants of AGE readouts are summarized in Figure 3.

**FIGURE 3:**
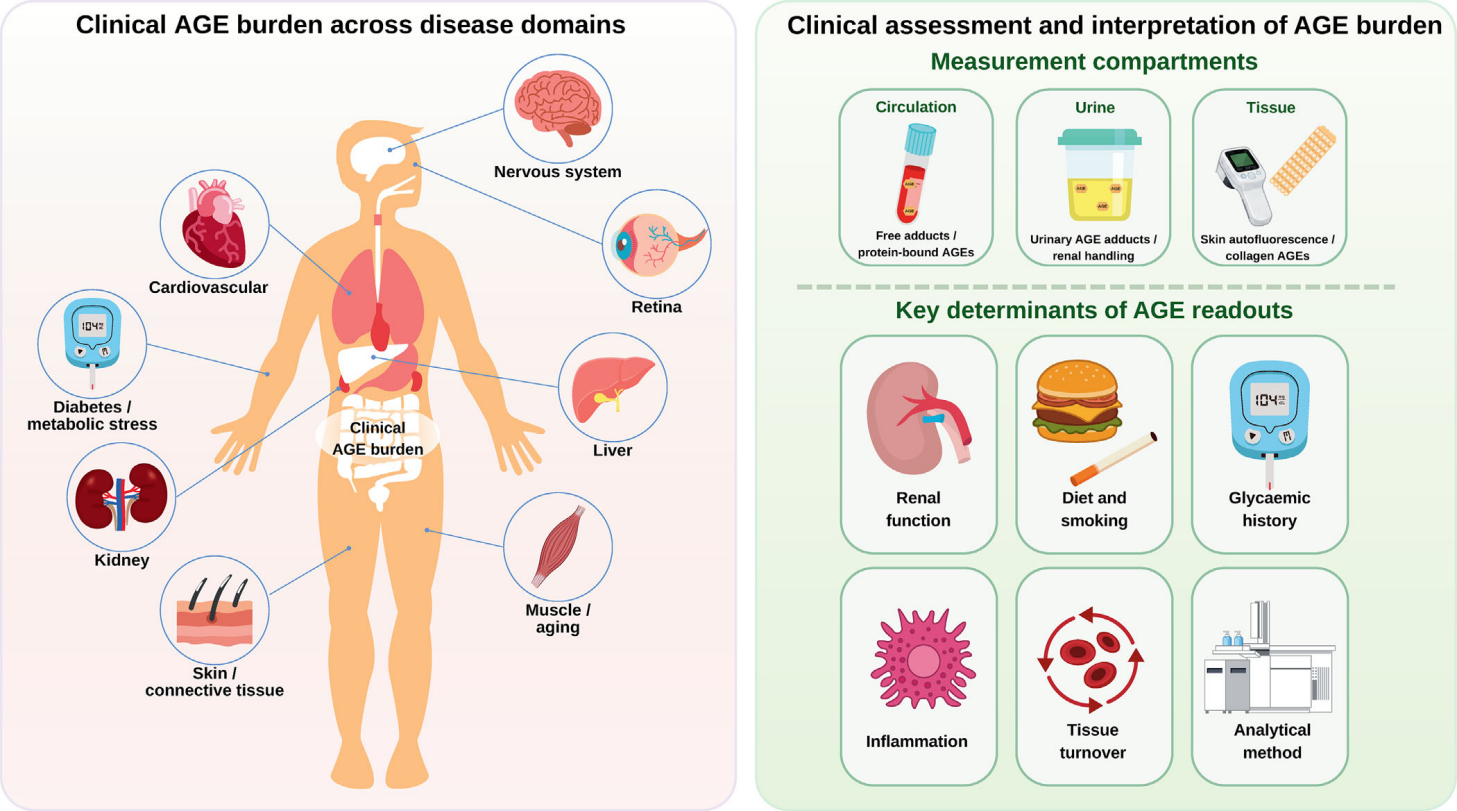
Clinical relevance and assessment of AGE burden. Integrated view of AGE burden across disease domains and clinical measurement compartments. AGE accumulation is clinically relevant to diabetes and metabolic stress, cardiovascular disease, kidney disease, neurovascular and retinal dysfunction, liver injury, muscle and aging‐related decline, and skin or connective‐tissue changes. Clinical assessment depends on the biological compartment being measured. Circulating samples capture free adducts and protein‐bound AGEs, urine reflects urinary AGE adducts and renal handling, and tissue‐level approaches such as skin autofluorescence or collagen AGE analysis provide surrogate information on longer‐term tissue accumulation. Interpretation of AGE readouts is influenced by renal function, diet and smoking, glycemic history, inflammation, tissue turnover, and analytical method. *Abbreviation*: AGE, advanced glycation end product.

## 5 Therapeutic Strategies: From Prevention to Intervention

Therapeutic strategies targeting AGE biology should be organized according to the stage of the AGE lifecycle they aim to modify. Upstream interventions reduce exogenous exposure or the metabolic conditions that drive endogenous formation. Carbonyl‐trapping agents and glyoxalase‐directed approaches target reactive dicarbonyl intermediates before stable AGE adducts accumulate. Clearance‐ and proteostasis‐directed strategies aim to reduce persistence of AGE‐modified products, whereas crosslink‐ and matrix‐directed approaches address established tissue retention and structural damage. RAGE‐directed therapies target the signaling arm of AGE biology, and natural compounds often act across multiple nodes with variable specificity. This section evaluates these strategies according to mechanism, evidence maturity, target engagement, and translational limitations. Importantly, no pharmacologic strategy discussed below is clinically established as an AGE‐targeted disease‐modifying therapy. Most remain preclinical or investigational, and the available clinical evidence is mixed or negative.

### 5.1 Upstream Prevention and Exposure Reduction

Upstream AGE prevention aims to reduce exogenous AGE exposure and limit the metabolic conditions that accelerate endogenous AGE formation. Dietary AGE reduction is the most direct exposure‐level strategy. AGE formation during cooking is promoted by high temperature, dry heat, prolonged heating, low water activity, and protein‐ or lipid‐rich food matrices; boiling, steaming, stewing, and poaching generally generate fewer AGEs than grilling, roasting, baking, broiling, or frying [38, 40]. Human intervention data support the feasibility of this approach, although the clinical effects remain variable. A systematic review of randomized controlled trials in adults with diabetes reported that low‐dietary AGE interventions reduced dietary AGE intake and affected several inflammatory, oxidative, lipid, and vascular‐related outcomes, while effects on glycemic indices were less consistent [176]. A randomized cross‐over feeding study using identical ingredients further showed that low‐AGE‐generating cooking methods, such as boiling and steaming, reduced serum AGEs and improved lipid‐related parameters compared with high‐AGE‐generating methods, such as grilling and baking [45]. These data support cooking‐method modification as a realistic exposure‐reduction strategy, while also indicating that biomarker improvement should not be assumed to translate automatically into durable clinical benefit.

Smoking cessation is a second upstream intervention because tobacco smoke contains reactive glycation products and carbonyl compounds that can modify circulating and structural proteins and perturb the RAGE axis [46, 177]. Reducing tobacco exposure therefore lowers an external source of carbonyl and AGE‐related burden, with broader vascular and inflammatory benefits [178, 179]. Metabolic optimization remains the foundation for reducing endogenous AGE formation. Sustained hyperglycemia increases early glycation reactions, whereas excess glycolytic flux and oxidative stress increase reactive dicarbonyl production, especially methylglyoxal [59]. Glycemic control, weight management, lipid control, blood‐pressure control, renal protection, and reduction of chronic inflammation may therefore reduce AGE burden indirectly by decreasing substrate pressure, carbonyl generation, oxidative stress, tissue injury, and impaired clearance [180]. These measures should be viewed as upstream risk‐modifying strategies rather than specific anti‐AGE therapies. Their main value is to reduce the biochemical environment that drives AGE formation and accumulation before long‐lived tissue proteins and extracellular matrices become extensively modified.

### 5.2 Pharmacologic Strategies Targeting AGE Biology

#### 5.2.1 Inhibition of AGE Formation or Carbonyl Trapping

Pharmacologic inhibition of AGE formation has mainly targeted reactive carbonyl intermediates before they modify proteins, lipids, or nucleic acids. Aminoguanidine, also known as pimagedine, was one of the earliest carbonyl‐trapping agents developed for this purpose [181, 182]. In experimental systems, it reduced dicarbonyl‐mediated AGE formation, but clinical translation was limited. In the ACTION I trial in Type 1 diabetic nephropathy, pimagedine did not significantly reduce the primary endpoint of time to doubling of serum creatinine, although it reduced proteinuria and slowed retinopathy progression. However, safety concerns, including glomerulonephritis at the higher dose, constrained further development [183]. Pyridoxamine, a vitamin B6 derivative, was developed as a broader inhibitor of advanced glycation and lipoxidation through carbonyl scavenging, metal chelation, and inhibition of oxidative steps in Amadori product degradation [184]. Combined Phase 2 studies suggested potential renal signals in selected patients with diabetic nephropathy, but a later randomized trial in Type 2 diabetic nephropathy did not establish a robust renoprotective effect across the overall study population [185, 186]. Thus, aminoguanidine was limited by both efficacy and safety concerns, whereas pyridoxamine has shown inconsistent renal efficacy; neither has an established clinical role as an AGE‐targeted therapy.

These trial histories do not invalidate carbonyl trapping as a therapeutic concept, but they clarify the translational requirements. Effective agents need adequate exposure in the relevant tissue compartment, selectivity for pathogenic dicarbonyls, long‐term safety, and pharmacodynamic evidence that methylglyoxal‐, glyoxal‐, or 3‐deoxyglucosone‐derived adducts are reduced in the target biological fraction. Emerging discovery strategies may help address this limitation. For example, deep‐learning‐based screening has recently been used to identify candidate methylglyoxal scavengers, including TP‐41, which showed preclinical activity in models of methylglyoxal‐associated memory impairment [187]. Such approaches remain early‐stage, but they illustrate a shift from broad antiglycation screening toward chemically defined dicarbonyl‐scavenger discovery. Without species‐specific target engagement, however, inhibition of AGE formation risks remaining an in vitro antiglycation effect rather than a disease‐modifying therapy.

#### 5.2.2 Enhancement of Detoxification or Clearance

Enhancing detoxification aims to strengthen endogenous systems that metabolize reactive carbonyls or remove AGE‐modified products. The glyoxalase pathway is the best‐defined target because GLO1 and GLO2 convert methylglyoxal‐derived glutathione hemithioacetal into d‐lactate while regenerating glutathione [56]. Since GLO1 acts upstream of methylglyoxal‐derived adduct formation, its therapeutic relevance should be assessed through methylglyoxal, MG‐H1, d‐lactate, glutathione status, and glyoxalase activity rather than through nonspecific AGE fluorescence alone [188]. Experimental studies show that GLO1 overexpression reduces hyperglycemia‐induced AGE formation and oxidative injury, while NRF2 directly regulates GLO1 transcription through an antioxidant‐response element, linking dicarbonyl detoxification with redox and electrophile‐stress defense [57, 60]. Clinical evidence for glyoxalase induction remains limited but informative. A trans‐resveratrol and hesperetin (tRES–HESP) combination was identified as a GLO1‐inducing formulation and evaluated in overweight and obese subjects, where it improved insulin resistance, dysglycemia, vascular function, and inflammatory markers in a randomized placebo‐controlled crossover study [189]. Subsequent analyses linked this intervention to methylglyoxal‐related target pharmacology and broader metabolic and inflammatory readouts [190]. More recent work suggests that tRES–HESP may regulate additional stress‐response genes beyond GLO1 in endothelial cells, fibroblasts, and HepG2 cells, supporting a broader dicarbonyl–redox–proteostasis defense profile [191]. These data provide a human proof‐of‐pharmacology signal, but they do not yet establish disease‐modifying efficacy in diabetic vascular, renal, neural, or fibrotic complications.

Clearance‐directed intervention is less mature. Renal filtration, tubular handling, proteostasis, autophagy, lysosomal degradation, and matrix turnover all influence AGE persistence, but few therapies selectively enhance AGE clearance in vivo. Current approaches are therefore mostly indirect, including preservation of kidney function, improvement of glutathione‐dependent detoxification, support of proteostatic capacity, and reduction of carbonyl input [192, 193]. Future studies should combine pharmacokinetic and pharmacodynamic readouts, including methylglyoxal levels, defined free and protein‐bound AGE adducts, glyoxalase activity, renal handling indices, and tissue AGE markers. Without these mechanism‐matched measurements, detoxification‐ or clearance‐enhancing strategies cannot be distinguished from broad antioxidant or metabolic interventions.

#### 5.2.3 Crosslink‐Directed Approaches

AGE‐crosslink and matrix‐targeted therapy addresses the long‐lived structural consequences of AGE accumulation. This strategy differs from carbonyl trapping or glyoxalase induction because it targets extracellular matrix damage after AGE‐modified collagen, elastin, or basement membrane proteins have already accumulated. Conceptually, three therapeutic directions should be separated: limiting new matrix crosslink formation, modifying established AGE‐stabilized crosslinks, and restoring matrix turnover or tissue compliance. This distinction is important because reducing dicarbonyl input may slow additional matrix damage, but it is unlikely to reverse decades of collagen crosslinking in vascular, renal, cardiac, hepatic, or connective tissues.

Alagebrium chloride, formerly ALT‐711, remains the best‐known clinical candidate in this category. Early studies reported improved arterial compliance in older adults with vascular stiffening and favorable signals in diastolic heart failure [194, 195]. However, later clinical evidence was less supportive. Alagebrium did not improve exercise tolerance or secondary cardiac endpoints in chronic heart failure, and a 1‐year intervention study in older individuals found no independent benefit of alagebrium on vascular function or enhancement of exercise‐training effects [196, 197]. These predominantly negative or inconclusive clinical findings argue against broad “AGE‐breaker” claims; alagebrium has no established clinical efficacy, and matrix glycation may be difficult to reverse once tissue architecture, fibrosis, and mechanical remodeling are advanced.

More recent work has shifted the field toward crosslink‐specific measurement and tissue‐level target validation. Glucosepane is a major collagen AGE crosslink in human tissues, but historically it has been difficult to quantify and directly target. New analytical tools, including antiglucosepane antibodies, enable more specific detection of tissue‐relevant AGE crosslinks [198]. Tissue‐level studies further support the need for this specificity. In scarred liver tissue, AGE‐mediated extracellular matrix crosslinking contributed to abnormal collagen architecture and impaired macrophage‐mediated matrix remodeling [18]. In a 2025 skeletal–muscle matrix study, AGE‐crosslinked decellularized muscle matrix reduced regenerative capacity and increased inflammatory markers in an in vivo volumetric muscle loss model [159]. These findings suggest that future crosslink‐directed therapies should be evaluated with direct matrix AGE measurements, mechanical readouts, degradability or remodeling assays, and tissue‐specific functional endpoints. Crosslink‐directed therapy remains plausible for vascular stiffening, myocardial fibrosis, renal fibrosis, liver fibrosis, diabetic connective‐tissue injury, and aging‐related matrix dysfunction, but clinical translation will require proof that specific AGE crosslinks are modified in the affected matrix compartment and that this biochemical change improves tissue mechanics or disease trajectory.

#### 5.2.4 Targeting AGE–RAGE Signaling

RAGE‐directed therapy aims to interrupt the conversion of AGE accumulation into sustained inflammatory, oxidative, vascular, and immune activation. Several approaches have been explored, including soluble RAGE or decoy‐based ligand sequestration, extracellular RAGE antagonism, inhibition of RAGE expression, downstream pathway blockade, and more recently disruption of intracellular RAGE signaling [79, 199, 200]. Recombinant soluble RAGE has shown protective effects in preclinical inflammatory and vascular models by binding RAGE ligands without initiating cell signaling, but clinical translation is constrained by delivery, pharmacokinetics, ligand promiscuity, and uncertainty over which disease states are truly RAGE driven [201]. Small‐molecule extracellular antagonism offers better drug‐like feasibility, but clinical experience has been disappointing. Azeliragon, an oral extracellular RAGE antagonist developed mainly for Alzheimer's disease [202], failed to demonstrate efficacy in the Phase 3 STEADFAST trial, which was terminated for lack of efficacy at the 5‐mg dose (NCT02080364). Extracellular RAGE blockade therefore remains clinically unvalidated as a disease‐modifying strategy. A more recent strategy targets the intracellular RAGE–DIAPH1 signaling interface rather than extracellular ligand recognition. The cytoplasmic domain of RAGE interacts with DIAPH1, and disruption of this interface reduced inflammatory, ischemic, renal, and wound‐healing complications in diabetic mouse models [19]. This approach is attractive because it targets a convergent signaling checkpoint downstream of multiple RAGE ligands while avoiding complete blockade of extracellular ligand recognition. Recent structural and functional work has strengthened this direction. RAGE406R, a small‐molecule antagonist of the RAGE–DIAPH1 interaction, inhibited DIAPH1 activation, suppressed actin polymerization and inflammatory responses in cellular models, reduced CCL2 expression in macrophages derived from individuals with Type 1 diabetes, and improved inflammatory and wound‐healing outcomes in mouse models [20]. This approach may provide greater signaling selectivity than ligand‐directed blockade, although it remains preclinical. Clinical translation will require tissue‐relevant pathway engagement, validated downstream biomarkers, appropriate disease timing, and safety evaluation, because RAGE is a multiligand pattern‐recognition receptor involved in inflammation, tissue injury responses, and host defense. Representative pharmacologic strategies targeting AGE formation, detoxification, clearance, matrix crosslinking, RAGE signaling, and downstream inflammatory pathways are summarized in Table 2.

**TABLE 2:** Pharmacologic strategies targeting AGE biology: mechanisms and translational status.

Strategy/target	Representative agents/platforms	Target/mode of action	Evidence status	Main limitation	References
AGE‐formation inhibition	Pimagedine/aminoguanidine	Reactive carbonyl trapping; reduced AGE formation	Preclinical: in vitro/in vivo Clinical: late‐stage RCT (Type 1 DN, ACTION I; no registry identifier reported) primary endpoint negative not approved	Safety and endpoint failure	[181, 182, 183]
Carbonyl trapping/antiglycation	Pyridoxamine/pyridorin	Dicarbonyl scavenging; Amadori oxidation inhibition	Preclinical: in vitro/in vivo Clinical: Phase 2 DN studies (PYR‐206, NCT00320060; PYR‐205/207, NCT00320021); later Phase 2b RCT (PYR‐210, NCT00734253) inconclusive; not approved.	Inconsistent renal efficacy	[184, 185, 186]
Dicarbonyl detoxification	Glyoxalase‐1 enhancement	MGO detoxification through increased glyoxalase flux	Preclinical: in vitro/in vivo Clinical: proof‐of‐pharmacology crossover RCT (tRES‐HESP; NCT02095873) no complication trial	Limited clinical endpoint data	[57, 58, 59, 60, 61, 189, 190, 191]
Redox‐linked detoxification	NRF2–GLO1 axis	GLO1 transcription; GSH‐linked dicarbonyls defense	Preclinical: in vitro/in vivo Clinical: indirect proof‐of‐pharmacology no disease‐endpoint trial	Pleiotropic pathway activation	[60, 189, 190, 191]
Clearance support	Renal and proteostatic handling	Free AGE adduct excretion; AGE–protein turnover	Preclinical: in vitro/in vivo Clinical: observational clearance/renal‐handling evidence no selective clearance therapy	Not AGE‐specific	[63, 64, 65, 87, 132, 133, 134]
AGE‐crosslink/matrix targeting	Alagebrium/ALT‐711	AGE‐modified ECM; crosslink modulation	Preclinical: in vivo Clinical: early arterial‐compliance study; DIAMOND (NCT00043836); BENEFICIAL (NCT00516646); older‐adult factorial RCT (NCT01417663); mixed/negative; not approved.	Uncertain tissue target engagement	[194, 195, 196, 197]
Glucosepane‐directed detection	Antiglucosepane antibodies	Matrix‐bound glucosepane detection	Analytical: in vitro/ex vivo tissue detection preclinical tool not therapeutic testing	Detection rather than therapy	[35, 198]
RAGE antagonism	Azeliragon/PF‐04494700	Extracellular RAGE blockade	Preclinical: in vitro/in vivo Clinical: Phase 3 AD development efficacy not established not approved (NCT02080364)	Disease and patient selection	[199, 200, 201, 202]
Decoy strategies	Soluble RAGE/sRAGE‐based approaches	RAGE ligand sequestration	Preclinical: in vitro/in vivo Clinical: biomarker/observational evidence only no therapeutic trial	Delivery and interpretation limits	[76, 77, 78, 79, 80, 201]
RAGE–DIAPH1 blockade	Small molecule antagonists	RAGE cytoplasmic signaling blockade	Preclinical: in vitro*/*ex vivo/in vivo Human ex vivo: macrophage target engagement no clinical trial	No clinical validation yet	[19, 20, 75, 99, 100, 200]
Downstream pathway blockade	NOX/NF‐κB/inflammatory signaling inhibitors	Redox‐inflammatory amplification control	Preclinical: in vitro/in vivo Clinical: indirect evidence only no AGE‐specific target‐engagement trial	Weak AGE‐specific target engagement	[92, 93, 94, 95, 96, 97, 98, 101, 102, 103, 104, 105, 106, 107, 118, 119]

Clinical trial registration identifiers are provided where available. No registry identifier was reported for ACTION I or the early ALT‐711 arterial‐compliance study.

*Abbreviations*: AGE, advanced glycation end product; DIAPH1, diaphanous‐related formin 1; ECM, extracellular matrix; GLO1, glyoxalase 1; GSH, reduced glutathione; MGO, methylglyoxal; NF‐κB, nuclear factor kappa B; NOX, NADPH oxidase; RAGE, receptor for advanced glycation end products; sRAGE, soluble receptor for advanced glycation end products.

### 5.3 Natural Compounds and Multitarget Interventions

#### 5.3.1 Reducing Dicarbonyl Stress and AGE Formation

Natural compounds have been widely studied as anti‐AGE agents, but their relevance should be judged by chemically defined activity rather than by broad antioxidant claims. Most evidence derives from cell‐free, cellular, or animal studies, while the limited human trials report biomarker or proof‐of‐pharmacology outcomes rather than established disease modification. At the formation stage, the most plausible mechanisms include trapping of reactive dicarbonyls, inhibition of oxidative Amadori‐product degradation, metal chelation, suppression of lipid peroxidation‐derived carbonyls, and protection of glycation‐prone protein sites [203, 204]. Polyphenols and phenolic acids remain the most frequently investigated classes. Classical model‐system studies showed that quercetin and genistein inhibit AGE formation by trapping methylglyoxal and glyoxal [205, 206], while recent glucose–lysine Maillard model work indicates that phenolic acid structure, including hydroxylation pattern, conjugation, and substituent chemistry, influences AGE‐generation inhibitory activity [204]. Clinical evidence is still limited, although a double‐blind randomized controlled trial in adults with central obesity showed the feasibility of testing naturally derived compounds such as lutein with defined AGE‐related outcomes [207]. Translation remains constrained by absorption, metabolism, conjugation, tissue distribution, and dose realism, because parent compounds active in protein–sugar or dicarbonyl‐trapping assays may not reach disease‐relevant compartments at active concentrations [208]. Future studies should therefore pair natural‐compound interventions with pharmacokinetic exposure data and chemically defined AGE readouts, such as MG‐H1s, CML, CEL, protein‐bound adducts, or tissue‐retained AGE markers. Without such target‐engagement evidence, natural compounds should be considered candidate modulators of dicarbonyl stress rather than established AGE‐lowering therapies.

#### 5.3.2 Suppressing AGE–RAGE Inflammatory Amplification

Natural compounds may also attenuate AGE‐associated inflammatory amplification, although their effects are usually pleiotropic rather than RAGE‐specific. Curcumin, sulforaphane, apigenin, baicalein, geniposide, crocin, and related phytochemicals have been reported to reduce RAGE expression, suppress AGE‐induced cytokine production, limit oxidative stress, or modulate downstream inflammatory and stress‐response pathways in cellular or animal models [209, 210, 211]. In d‐galactose‐induced aging rats, apigenin and baicalein improved thoracic aortic structural deterioration and cognitive deficit in association with inhibition of the AGE–RAGE/NF‐κB pathway [212]. Geniposide was reported to ameliorate diabetic nephropathy in Type 2 diabetic mice by targeting an AGE–RAGE‐dependent inflammatory pathway, with evidence that geniposide competitively interfered with AGE–RAGE interaction through binding to the RAGE V‐domain [213]. Crocin has also been examined in diabetic tissue injury models, including studies reporting modulation of RAGE, TNF‐α, and TGF‐β expression in diabetic rats [214]. Together, these examples indicate an anti‐inflammatory signal, but they do not establish RAGE‐specific therapeutic action. The key distinction is whether inflammation declines because AGE–RAGE signaling has been directly modified, or because the compound broadly improves carbonyl stress, redox balance, metabolism, mitochondrial function, or cytoprotection. Stronger evidence will require defined AGE species, receptor‐pathway engagement, pharmacokinetic exposure, and disease‐relevant functional outcomes to be measured in the same experimental framework.

#### 5.3.3 Preserving Protein, Matrix, and Barrier Integrity

Natural compounds may also protect tissue integrity by limiting AGE‐associated protein aggregation, matrix crosslinking, endothelial dysfunction, and epithelial or endothelial barrier injury. This direction is relevant because AGE pathology includes long‐lived protein modification, collagen crosslinking, matrix resistance to degradation, and failure of tissue repair. Chlorogenic acid inhibited AGE formation and AGE‐associated protein crosslinking in vitro, supporting a matrix‐protective mechanism at the level of early glycation and crosslink generation [215]. Rosmarinic acid provides a more tissue‐oriented example: in scarred liver tissue, AGE‐mediated extracellular matrix crosslinking contributed to abnormal matrix architecture and impaired macrophage‐mediated remodeling, while rosmarinic acid inhibited AGE‐mediated crosslinking and alleviated fibrosis progression in experimental models [18]. Formulation‐enabled strategies may further improve tissue targeting. An echinacoside–zinc nanomaterial inhibited skin glycation and suppressed RAGE expression through regulation of the MDM2/STAT2/RAGE axis, providing an example of a delivery‐based antiglycation approach [216].

Collectively, these examples shift natural‐compound discussion beyond soluble antiglycation activity toward tissue preservation. Many compounds discussed in this section, such as quercetin, curcumin, resveratrol, genistein, sulforaphane, and chlorogenic acid, occur in commonly consumed plants, spices, and beverages, including resources with histories of both dietary and medicinal use. This overlap gives the food–medicine homology framework particular translational potential, because selected edible resources may provide nutritional value together with multiple bioactive constituents with the potential to modulate AGE formation, AGE–RAGE inflammatory amplification, and tissue injury [217]. In practice, this could combine lower‐AGE food selection and cooking methods with the inclusion of phytochemical‐rich foods as part of a sustained dietary strategy. Realizing this potential, however, requires evidence that achievable dietary intake produces sufficient bioavailability and target engagement in disease‐relevant tissues, because whole‐food exposure cannot be inferred from pharmacological studies of isolated compounds. Claims of protein, matrix, or barrier protection should therefore remain mechanism‐linked unless supported by compartment‐specific AGE measurements, tissue exposure data, and functional outcomes such as matrix degradability, stiffness, permeability, fibrosis, or repair capacity. Representative natural compounds and multitarget anti‐AGE interventions, together with their proposed mechanisms and evidence maturity, are summarized in Table 3.

**TABLE 3:** Natural compounds and multitarget anti‐AGE interventions: representative mechanisms and evidence maturity.

Compound class/intervention	Representative examples	AGE‐related action	Main model/context	Evidence level	References
Dietary polyphenols	Flavonoids; phenolic acids; stilbenes; tannins; mixed plant polyphenols	Carbonyl trapping; antioxidant protection; AGE–RAGE modulation	Maillard systems; protein‐glycation assays; AGE‐treated cells; diabetic rodents	Preclinical: cell‐free/in vitro/cellular/in vivo; clinical evidence compound‐specific	[203, 208]
Flavonols	Quercetin; rutin; kaempferol; myricetin	MGO/GO trapping; reduced AGE formation	Cell‐free MGO/GO trapping assay; physiological pH 7.4, 37°C	Cell‐free/in vitro; limited translational evidence	[203, 205, 208]
Isoflavones	Genistein; daidzein; soy isoflavones	MGO trapping; antiglycation activity	Cell‐free MGO/GO trapping assay; physiological pH 7.4, 37°C	Cell‐free/in vitro; limited disease‐model evidence	[203, 206, 208]
Flavones/flavonoids	Apigenin; baicalein; luteolin; chrysin	AGE–RAGE/NF‐κB suppression	d‐Galactose‐induced aging rats; thoracic aorta and cognitive outcomes	Preclinical in vivo; no AGE‐specific clinical trial	[203, 208, 212]
Catechins/tea polyphenols	Catechin; epicatechin; EGCG; green tea polyphenols	Carbonyl trapping; antioxidant protection; reduced AGE formation	BSA‐glucose; BSA‐MGO; Maillard reaction systems	Cell‐free/in vitro; limited disease‐specific in vivo evidence	[203, 208]
Phenolic acids	Chlorogenic acid; caffeic acid; ferulic acid; gallic acid; ellagic acid; rosmarinic acid	Reduced AGE formation; protein or matrix crosslinking inhibition	Glucose‐lysine Maillard model; BSA‐glucose glycation; AGE‐collagen crosslinking; scarred liver fibrosis model	Cell‐free/in vitro; preclinical in vivo liver‐fibrosis support	[18, 203, 204, 215]
Stilbenes	Resveratrol; pterostilbene; tRES‐HESP formulation	Antioxidant protection; AGE–RAGE pathway modulation; GLO1 induction	AGE‐treated vascular cells; diabetic animal models; overweight/obese adults (tRES‐HESP; NCT02095873)	Preclinical in vitro/in vivo; human proof‐of‐pharmacology RCT; no complication trial	[189, 190, 191, 203, 208]
Curcuminoids	Curcumin; demethoxycurcumin; bisdemethoxycurcumin	AGE receptor regulation; inflammatory signaling control	AGE‐treated hepatic stellate cells; leptin‐signaling context	Preclinical cellular/in vitro; no clinical AGE‐endpoint trial	[203, 208, 209]
Carotenoids	Lutein; zeaxanthin; carotenoid‐rich extracts	Reduced AGE‐related burden; antioxidant protection	Adults with central obesity; 32‐week double‐blind randomized controlled trial	Human RCT with AGE‐related readouts (ChiCTR1800018098); not disease‐endpoint trial	[207, 208]
Apocarotenoids	Crocin; crocetin	RAGE/TGF‐β/TNF‐α modulation	Streptozotocin‐induced diabetic male Wistar rats; liver tissue	Preclinical in vivo; no clinical AGE‐specific validation	[208, 214]
Isothiocyanates/NRF2 activators	Sulforaphane; broccoli‐derived isothiocyanates	Reduced AGE‐induced inflammation and RAGE expression; NRF2‐linked cytoprotection	AGE‐treated endothelial cells and rat aorta; AGE‐treated pericytes	Preclinical in vitro/ex vivo/in vivo; no clinical AGE‐endpoint trial	[210, 211]
Iridoids/terpenoids	Geniposide; iridoid glycosides; selected terpenoids	AGE–RAGE inflammatory pathway inhibition	Diabetic nephropathy mouse model	Preclinical in vivo; no clinical trial	[208, 213]
Natural product formulations	Echinacoside–zinc nanomaterial; polyphenol‐based formulations; plant‐extract formulations	RAGE transcriptional suppression; antiglycation effect	MGO‐induced HaCaT keratinocyte model; high‐fat diet/d‐galactose skin‐glycation mouse model	Preclinical in vitro/in vivo; formulation‐stage evidence	[208, 216]

*Evidence‐level classification*: Cell‐free and cellular studies are considered subclasses of in vitro evidence; “cell‐free” refers to chemical reaction systems or purified biomolecules, whereas “cellular” refers to cultured‐cell models. Ex vivo evidence refers to isolated cells or tissues studied outside the intact organism, and in vivo evidence refers to studies conducted in intact animals. Human interventional evidence refers to studies in which an intervention was administered to human participants; proof‐of‐pharmacology or biomarker trials were distinguished from disease‐endpoint trials. “Preclinical” encompasses cell‐free, cellular, ex vivo, and animal evidence. Where evidence spans multiple categories, the applicable levels are listed together. These categories describe experimental setting and translational maturity and do not constitute a formal assessment of study quality or risk of bias.

*Abbreviations*: AGE, advanced glycation end product; EGCG, epigallocatechin gallate; GO, glyoxal; HO‐1, heme oxygenase‐1; MGO, methylglyoxal; NF‐κB, nuclear factor kappa B; NRF2, nuclear factor erythroid 2‐related factor 2; RAGE, receptor for advanced glycation end products; TGF‐β, transforming growth factor beta; TNF‐α, tumor necrosis factor alpha.

### 5.4 Translational Constraints Across Anti‐AGE Therapies

The current bottleneck in anti‐AGE therapy is no longer biological plausibility, but the mismatch between therapeutic mechanism, disease stage, and clinical endpoint. Anti‐AGE strategies fail to translate when interventions that act on different compartments are judged by the same generic AGE readout. Dietary modification mainly reduces exogenous exposure; carbonyl scavengers should intercept reactive dicarbonyl intermediates; glyoxalase‐directed approaches should improve methylglyoxal handling; matrix‐targeted strategies should address established tissue crosslinks; RAGE‐directed therapies should suppress signaling amplification; and natural compounds often act across several nodes with uncertain tissue exposure. Each strategy therefore requires a mechanism‐matched definition of success. A low‐AGE dietary intervention may lower circulating AGE‐related signals without reversing tissue‐retained matrix damage; a carbonyl scavenger should reduce defined dicarbonyl‐derived adducts; a glyoxalase inducer should improve methylglyoxal detoxification; a crosslink‐directed therapy should modify tissue‐relevant matrix crosslinks or mechanics; and a RAGE‐directed therapy should show pathway‐level engagement. The 2025 cooking‐method trial illustrates this distinction: low‐AGE‐generating methods reduced serum AGEs and improved lipid‐related parameters, but whether such biomarker changes translate into durable cardiometabolic or organ‐protective benefit remains unresolved [45].

A second constraint is disease timing and patient selection. Anti‐AGE strategies are unlikely to have equal value across early dicarbonyl stress, established extracellular matrix crosslinking, advanced fibrosis, renal failure, and late degenerative tissue damage. At each stage, the dominant pathogenic compartment should determine both the therapeutic target and the population selected for treatment. Formation‐ or detoxification‐directed interventions are most plausible before irreversible tissue damage becomes established, whereas matrix‐directed strategies are more relevant to accumulated tissue crosslinks and RAGE‐directed therapy may be better suited to an AGE/RAGE‐active inflammatory phenotype than to unselected chronic disease populations. In early diabetic kidney disease with preserved or mildly reduced filtration but elevated methylglyoxal or MG‐H1, a carbonyl scavenger or GLO1 inducer could target circulating and renal dicarbonyl stress. Reductions in plasma methylglyoxal or protein‐bound MG‐H1, together with increased GLO1 activity where applicable, would demonstrate target engagement, while albuminuria and estimated glomerular filtration rate decline would provide renal endpoints [10]. In patients with established arterial stiffness and high tissue AGE burden, a matrix‐directed intervention would instead target long‐lived vascular collagen. Skin autofluorescence could support patient enrichment, whereas direct collagen‐crosslink measurement in a biopsy substudy would provide stronger evidence of matrix target engagement, with carotid–femoral pulse‐wave velocity or vascular compliance as functional endpoints [121, 127]. In chronic diabetic wounds with an AGE–RAGE–DIAPH1 inflammatory signature, a RAGE–DIAPH1 inhibitor would target signaling in wound macrophages and endothelial cells. Suppression of pathway‐linked mediators such as CCL2 in wound tissue or exudate could provide pharmacodynamic evidence, while time to complete wound closure or the proportion of ulcers healed within a prespecified period would provide clinical endpoints [19, 20]. Each design therefore specifies the disease stage, target compartment, pharmacodynamic biomarker, and clinical endpoint rather than relying on a generic change in “total AGEs.” These examples are proposed trial designs rather than established clinical approaches and will require pathway biomarkers relevant to the affected tissue, safety evaluation, and patient stratification before clinical testing.

Natural compounds and emerging antiglycation candidates face an additional exposure problem. Many agents show activity in protein–sugar systems, dicarbonyl‐trapping assays, AGE‐treated cells, or diabetic rodents, but the active parent compound may be poorly absorbed, extensively metabolized, rapidly conjugated, or absent from the target tissue at effective concentrations [208]. Recent chemical‐biology work further emphasizes that protein glycation is chemically diverse, and that reducing bulk fluorescence or total AGE immunoreactivity does not necessarily modify the pathogenic species responsible for tissue injury [21]. Across all anti‐AGE strategies, future studies should therefore follow a stricter translational sequence: define the target AGE species or signaling pathway, demonstrate exposure in the relevant compartment, confirm pharmacodynamic target engagement, and link biochemical change to tissue function or clinical outcome. Without this sequence, negative trials may reflect inappropriate assay selection, inadequate tissue exposure, poor disease timing, or irreversible tissue damage rather than true irrelevance of AGE biology. The therapeutic landscape of AGE‐targeted intervention, from upstream prevention to mechanism‐matched pharmacologic and multitarget strategies, is summarized in Figure 4.

**FIGURE 4:**
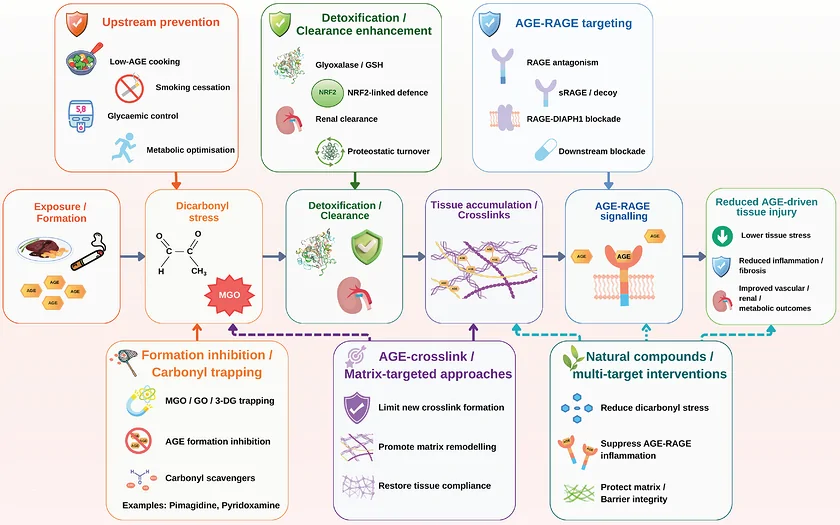
Integrative summary of the AGE lifecycle and mechanism‐matched therapeutic strategies. The schematic links major determinants of AGE burden to stage‐specific intervention opportunities. Upstream strategies reduce exogenous exposure and endogenous formation through low‐AGE cooking practices, smoking cessation, glycemic control, and metabolic optimization. Formation inhibitors and carbonyl scavengers intercept reactive dicarbonyls, including MGO, GO, and 3‐DG, before stable AGE formation. Enhancement of glyoxalase/GSH‐dependent detoxification, NRF2‐regulated defense, renal clearance, and proteostatic turnover supports AGE detoxification and removal. Matrix‐directed approaches aim to limit new crosslink formation, promote matrix remodeling, or restore tissue compliance. Receptor‐directed strategies target AGE–RAGE signaling through RAGE antagonism, sRAGE or other decoy approaches, RAGE–DIAPH1 blockade, and inhibition of downstream pathways. Natural compounds may act at multiple stages by reducing dicarbonyl stress, attenuating AGE–RAGE‐mediated inflammation and preserving matrix or barrier integrity. Clinical translation requires alignment of each intervention with the predominant AGE species, biological compartment, disease stage, and clinically relevant functional endpoint. *Abbreviations*: 3‐DG, 3‐deoxyglucosone; AGE, advanced glycation end product; DIAPH1, diaphanous‐related formin 1; GO, glyoxal; GSH, reduced glutathione; MGO, methylglyoxal; NRF2, nuclear factor erythroid 2‐related factor 2; RAGE, receptor for advanced glycation end products; sRAGE, soluble receptor for advanced glycation end products.

## 6 Conclusion and Prospects

### 6.1 AGE Burden as a Dynamic Pathogenic System

AGEs should be understood as a dynamic pathogenic system rather than as passive metabolic residues or a single class of interchangeable biomarkers. Their biological relevance arises from the balance among endogenous formation, exogenous exposure, dicarbonyl detoxification, protein turnover, tissue retention, and renal clearance. Diabetes provides the clearest clinical model because sustained hyperglycemia and methylglyoxal‐driven dicarbonyl stress accelerate AGE formation, while long‐lived tissue proteins and extracellular matrices preserve glycation‐derived injury over time. The pathogenic effects of AGEs operate through two interconnected routes: receptor‐independent damage, including protein dysfunction, impaired proteostasis, extracellular matrix crosslinking, altered tissue mechanics, and reduced matrix degradability; and receptor‐dependent signaling, mainly through RAGE and related pathways that convert AGE accumulation into sustained redox, inflammatory, vascular, and immune activation. Chronic AGE pathology is best viewed as a layered process in which soluble dicarbonyl stress, tissue‐retained matrix damage, receptor‐mediated inflammatory amplification, impaired clearance, and disease‐specific tissue vulnerability reinforce one another. This systems‐level view is more clinically useful than the traditional AGE–RAGE–oxidative stress model because it explains why AGE biology differs across diabetes, cardiovascular disease, kidney disease, neurodegeneration, fibrosis, and aging‐related tissue dysfunction.

### 6.2 Unresolved Conceptual Challenges

The central conceptual challenge is not simply AGE heterogeneity, but causal prioritization. AGE species differ in precursor chemistry, molecular target, tissue distribution, turnover, biological activity, and detectability. Therefore, they cannot be treated as equivalent readouts of the same pathological process. A circulating free adduct may indicate recent formation, dietary absorption, proteolytic release, or reduced renal clearance, whereas a matrix‐bound collagen crosslink may represent years of tissue retention and mechanical damage. Similarly, AGE–RAGE signaling can explain inflammatory amplification, but it does not fully account for established matrix stiffening, proteostatic failure, or long‐lived protein dysfunction. Future AGE biology must therefore ask more selective questions: which AGE species accumulates, where it acts, whether it is reversible, which cells respond to it, and whether modifying it changes tissue function. Without this causal hierarchy, AGE research risks remaining chemically sophisticated but clinically ambiguous.

### 6.3 Methodological and Translational Priorities

The next phase of AGE research should move from isolated AGE measurements toward longitudinal, multicompartment study designs. A stronger framework would combine species‐defined LC–MS/MS profiling, plasma and urinary free‐adduct measurements, protein‐bound AGE assessment, tissue‐level readouts such as skin autofluorescence or matrix AGE analysis, renal handling indices, inflammatory or RAGE‐pathway markers, imaging, and functional tissue outcomes within the same cohorts [3, 21, 162]. This approach would allow investigators to distinguish recent dicarbonyl stress from long‐term tissue retention, impaired renal clearance, dietary exposure, and disease‐related matrix modification. It would also support more rigorous patient stratification by identifying AGE‐active phenotypes rather than treating all patients with diabetes, kidney disease, vascular stiffening, or aging‐related dysfunction as biologically equivalent. Emerging biosensors, point‐of‐care assays, machine‐learning models, and AI‐assisted risk tools may become useful in this setting if they integrate AGE species, renal function, glycemic history, diet, smoking, imaging, omics, medication exposure, and clinical trajectories into interpretable risk patterns. However, these technologies should be used as analytical accelerators rather than replacements for chemical validation. The field will progress faster if computational tools are trained on standardized AGE assays, tested in longitudinal datasets, and linked to tissue‐specific outcomes rather than used to generate broad AGE‐risk scores without mechanistic anchoring.

### 6.4 From Associative Biomarkers to Testable AGE Models

The most realistic near‐term progress will come from biomarker‐linked intervention studies in populations with high AGE burden and measurable disease trajectories. Diabetes, cardiorenal disease, vascular stiffening, fibrotic tissue remodeling, and selected aging‐related phenotypes are suitable starting points because AGE formation, tissue retention, renal handling, and clinical progression can be assessed longitudinally. The field should move away from asking whether “AGEs are increased” in disease and instead determine how formation, exposure, detoxification, clearance, tissue retention, and receptor signaling combine within individual patients. We hypothesize that clinically relevant AGE burden is a dynamic biological state that cannot be represented adequately by a single concentration. This hypothesis could be examined using a standardized AGE handling assessment in which a chemically defined meal is followed by serial measurement of specific AGE species in plasma and urine, together with glyoxalase activity, renal filtration and excretion, AGE adducts bound to proteins, tissue retention, and cellular responses to RAGE signaling through DIAPH1. These measurements could reveal whether AGE burden is driven mainly by increased formation or exposure, limited detoxification, impaired clearance, established tissue retention, or active receptor signaling. We predict that this integrated profile would identify patients at risk of tissue accumulation and disease progression more accurately than circulating AGEs, urinary AGE excretion, or skin autofluorescence considered separately.

Overall, this review argues that AGEs should no longer be treated as a single pathogenic entity, a single biomarker class, or a single therapeutic target. AGE burden is a systems‐level process shaped by formation, exposure, detoxification, clearance, and tissue retention, and its consequences arise through both receptor‐independent structural damage and receptor‐dependent inflammatory amplification. Diabetes remains the strongest clinical anchor, but AGE biology extends across cardiovascular, renal, neural, retinal, fibrotic, and aging‐related disease contexts. The next phase should test whether therapeutic response can be predicted by matching treatment to the dominant AGE source, compartment, mechanism, and disease stage. Strategies that reduce dietary exposure or dicarbonyl formation may be most effective when exposure or endogenous formation is the principal driver, whereas impaired clearance, established matrix accumulation, or persistent RAGE signaling may require interventions that reach the affected tissue or pathway. One testable example is early diabetic kidney disease in patients with elevated methylglyoxal or MG‐H1 and evidence of active renal RAGE signaling. We hypothesize that combining a dicarbonyl scavenger with a RAGE–DIAPH1 inhibitor will provide greater benefit than either treatment alone by limiting new AGE formation while suppressing inflammatory responses to residual AGE ligands. A randomized factorial trial could stratify patients according to their baseline AGE profiles, quantify plasma methylglyoxal and MG‐H1, assess a prespecified RAGE–DIAPH1 pharmacodynamic signature in urinary cells or circulating monocytes, and measure albuminuria and estimated glomerular filtration rate slope. Evidence of synergy would require a statistically significant interaction between the two treatments for a prespecified renal endpoint, together with engagement of both intended pathways. More broadly, future trials should classify patients by their AGE profile, verify engagement of the intended species, compartment, or pathway, and relate that engagement to recovery of tissue function or a change in disease trajectory. The field will advance when it can identify a defined component of AGE biology as causal, modify it in the relevant compartment, and demonstrate a corresponding improvement in tissue function or clinical progression.

## Author Contributions

*Review design and revision*: Tak‐Ho Lo and Mengyao Wu. *Manuscript writing*: Tak‐Ho Lo and Junying Zou. *Data collection*: Junying Zou, Jingyi Zhang, Kaijun Lin, Minhui Zhu, Shuangqi Lu, and Xin Liu. *Figure preparation*: Mengyao Wu. All authors have read and approved the final manuscript.

## Funding

This work was supported by the National Natural Science Foundation of China (32502104), Henan Province Science and Technology Key Research Project (262102310249), and the Research Fund Program of the Guangdong Provincial Key Laboratory of Speed Capability Research (2023B1212010009).

## Ethics Statement

The authors have nothing to report.

## Conflicts of Interest

The authors declare no conflicts of interest.

## Data Availability

No new data were generated or analyzed for this review.
